# Diversity and distribution of acrobat ants, *Crematogaster* Lund, 1831 (Formicidae, Myrmicinae), in the Colombian tropical dry forest

**DOI:** 10.3897/BDJ.14.e176466

**Published:** 2026-04-27

**Authors:** Daniela M. Gutiérrez-Martínez, Lina M. Ramos-Ortega, Roberto J. Guerrero

**Affiliations:** 1 Universidad del Magdalena, Santa Marta, Colombia Universidad del Magdalena Santa Marta Colombia

**Keywords:** rboreal ants, biological collections, heterogeneous distribution, Neotropics, taxonomy

## Abstract

**Background:**

The tropical dry forest (TDF) in Colombia is a highly threatened ecosystem by deforestation, intensive agriculture, urbanization and climate change. These pressures have caused extensive habitat loss and fragmentation, negatively impacting biodiversity and the ecological roles of key groups such as ants. Currently, the TDF is divided into six regions (Caribbean, Norandina>, Orinoquía, Patía River Valley, Cauca River Valley and Magdalena River Valley), based on the composition and other attributes of the vegetation that structures each of them. We document the species composition, richness and distribution of acrobat ants (*Crematogaster* Lund, 1831) in Colombian TDF, based on data from five regions (except the Norandina> region). Records were compiled from biological collections, project loans and standardized field sampling using mini-Winkler extractors, pitfall traps and manual collection.

**New information:**

In total, 23 species were recorded, with the Caribbean Region exhibiting the highest richness and the Patía River Valley the lowest. Species composition amongst regions was analyzed using non-metric multidimensional scaling (nMDS), based on the Jaccard similarity index, with subsequent permutational multivariate analysis of variance. The non-metric multidimensional scaling ordination, based on Jaccard dissimilarity, provided an adequate representation of the compositional relationships amongst sampling units across Colombian dry forest regions. PERMANOVA detected a significant overall effect of region on sampling unit composition. Data source also had a significant effect on sampling unit composition. Seven species were widely distributed, with *C. curvispinosa* being the most widespread, whereas six species were restricted to a single region, mainly the Caribbean. Our findings highlight the adaptability of *Crematogaster* to habitat fragmentation in TDF. However, gaps in geographic coverage likely underestimate true diversity, with important implications for conservation strategies targeting this threatened biome.

## Introduction

The tropical dry forest (TDF) biome extends across more than 50% of the tropical land surface in Africa, the Americas, Asia and parts of the Pacific (Murphy and Lugo 1986; Janzen 1988), based on analyses of climatic datasets (Ocón et al. 2021) that underpin widely applied bioclimatic definitions of TDF (Romero-Duque et al. 2019, Mesa-Sierra et al. 2025). Tropical dry forests are generally characterized by a pronounced dry season lasting up to three months, mean annual temperatures exceeding 26.5°C and average annual precipitation of approximately 1,575 mm (Pizano et al. 2014a, González-M et al. 2020).

Seasonality is a defining feature of TDFs and distinguishes them from other tropical forest biomes, particularly in terms of vegetation structure and phenology (Romero-Duque et al. 2019). Plant communities have evolved a wide range of adaptive strategies to cope with extended dry periods (Banda et al. 2016, Singh and Chaturvedi 2017), resulting in heterogeneous landscapes with temporally variable resources. These conditions support diverse and often unique animal assemblages (Hasnat and Hossain 2022) and TDFs generally exhibit higher levels of endemism and beta diversity than other tropical biomes (Dinerstein et al. 2017).

Despite broadly similar climatic constraints, TDFs show substantial regional variation in edaphic conditions, biological diversity, ecosystem functioning and the ecosystem services they provide (Thakur et al. 2021) , including climate regulation, carbon storage, erosion control, maintenance of soil quality, water supply and nutrient cycling (Quijas et al. 2019). The intensive exploitation of these ecosystem services — particularly because TDF distribution overlaps extensively with areas suitable for agriculture and livestock production — has made tropical dry forests one of the most threatened biomes worldwide (Miles et al. 2006, Mesa-Sierra et al. 2025).

Across the Neotropics, TDF extends from north-western Mexico to northern Argentina and south-eastern Brazil (Pizano et al. 2014a). In Colombia it once covered ~ 8 million ha, but now occupies only ~ 1,022,632 ha (González-M. et al. 2019), distributed in six regions: the Caribbean Region, the Magdalena and Cauca River valleys, the Patía River Valley, the Norandina> Region and the Orinoquía Region (González-M et al. 2020). Deforestation, agricultural expansion and mining have severely fragmented and degraded this ecosystem (Murphy and Lugo 1986, IAvH 1998, Pizano et al. 2014a, PNUD 2019) resulting in TDF fragments that potentially serve as important, but precarious biodiversity refuges. Ants, a conspicuous component of terrestrial ecosystems, are amongst the organisms most affected (Achury et al. 2012). Loss of natural habitat and vegetation negatively impacts arboreal ants, such as *Crematogaster*, a genus notable for its abundance, diversity and influence on ecosystem structure and function, making these ants valuable indicators for conservation and for monitoring threatened TDF habitats.

Ants of the genus *Crematogaster* (Formicidae, Myrmicinae), commonly known as “acrobat ants” (Blaimer 2012a), are diverse and widely distributed (Janicki et al. 2016, Guénard et al. 2017, Bolton 2025). Species of this genus are highly territorial and dominant in arboreal ant assemblages (Blaimer 2012a, Blaimer and Fisher 2013), occupying habitats from ground level to the forest canopy in ecosystems such as forests, jungles and savannahs. They exploit a broad range of nesting resources — including branches, living or decaying trunks and floral domes — demonstrating remarkable ecological flexibility (Hölldobler and Wilson 1990, Longino 2003, Blaimer 2010, Blaimer 2012b). *Crematogaster* ants rank amongst the most ecologically significant ant groups worldwide, strongly influencing the structure and functioning of terrestrial ecosystems, particularly in tropical and lowland regions.

In the Neotropical Region, Longino (2003) reviewed the genus *Crematogaster* in Costa Rica, describing 11 new species and reporting a total of 31 species for the country. In Colombia, Pedraza and Fernández (2019) recorded 27 species and updated their distribution. Although these studies addressed the diversity of the genus at a regional scale, several works have focused specifically on Colombian TDF. Álvarez et al. (1997) identified *Crematogaster* as one of the most representative genera in TDF remnants of the Caribbean Region and Achury et al. (2008) highlighted its role in studies of species composition and competitive interactions in Valle del Cauca. Simanca and Martínez (2010) reported *Crematogaster* as the most diverse genus in the Luriza Reserve, while Simanca et al. (2013) and Garzón and Chacón-De-Ulloa (2014) documented it as the most abundant in disturbed forest fragments and in Valle del Cauca, respectively. More recently, Ramos-Ortega et al. (2022) found that *Crematogaster* represented over 50% of ant species in TDF fragments of Santa Marta, with *C. crinosa* amongst the most abundant species and Sierra and Castrillo (2023) demonstrated ecological interactions of *C. crinosa* and *C. obscurata* with their plant hosts, where the ants protect plants in exchange for food and shelter.

The ant fauna inhabiting the Colombian dry forest (TDF) is currently poorly understood, with studies limited to local areas (Armbrecht and Chacón-de-Ulloa 1999, Achury et al. 2008, Domínguez et al. 2008, Diaz et al. 2009, Ramos-Ortega et al. 2022, Bautista-Giraldo et al. 2025) within some of the regions addressed here. These studies suggest a greater richness of ant species than expected, supporting the idea that some ant groups have successfully adapted to the ecological limitations of the dry forest. Specifically, *Crematogaster* ants appear to be one of the most representative groups of the dry forest in Colombia; however, the records come from local-scale studies, limiting the understanding of its diversity and the contribution of its species to the functionality of the ecosystem. The aim of this study was to document the composition and distribution of *Crematogaster* species in this ecosystem. To achieve this, we established the taxonomic identity of the species and mapped the identified species across the defined TDF regions. Furthermore, we assessed whether the distribution of *Crematogaster* species aligns with the geographic regionalization of the TDF in Colombia. Given the generalist nesting and foraging ecology of *Crematogaster* ants and their high dispersal ability, we hypothesize that assemblages inhabiting Colombian tropical dry forests are weakly structured by floristic regionalization, resulting in low compositional differentiation amongst regions despite strong habitat fragmentation within each region. Alternatively, if landscape fragmentation and historical–ecological constraints limit dispersal and establishment, *Crematogaster* assemblages are expected to show spatial segregation consistent with plant-based regionalization patterns. In this context, we assess the diversity, distribution and regional turnover of *Crematogaster* species across Colombian TDFs.

## Materials and methods

### Specimen processing

This study was conducted in the Tropical Dry Forest (TDF) of Colombia, which is divided into six main regions (Table 1). Five of these regions (Caribbean, CAR; Orinoquía, ORI; Patía River Valley, PRV; Cauca River Valley, CRV; and Magdalena River Valley, MRV) were included in this study, since public order problems did not allow us to obtain data from the Norandina> Region.

The data for this study were obtained from three sources: (1) fieldwork in several regions of tropical dry forest (hereafter STD); (2) examination of specimens deposited in national biological collections and museums (hereafter COL) and (3) specimens loaned or donated by other research projects (hereafter OTH). In the latter case, we validated the taxonomic identification of *Crematogaster* specimens previously reported by Ramos-Ortega et al. (2022), Guerrero et al. (2023) and Bautista-Giraldo et al. (2025). Ant sampling was conducted in TDF plots located in the Orinoquía and Caribbean regions of Colombia. The Caribbean Region harbours the largest extent of TDF in the country; therefore, seven sampling sites were established there, while only one site was sampled in the Orinoquía. In both regions, three complementary collection methods were employed: mini-Winkler extractors (30 samples) for leaf-litter ants, pitfall traps (30 units) for ground-dwelling ants and manual collection for vegetation-associated species, such as *Crematogaster*. At each site, three transects of 100 m each were established, with sampling station every 10 m to ensure structured coverage. For the mini-Winkler method, leaf litter was sifted and the fine material was processed in the field for 48 h. Pitfall trapping involved plastic cups (7 ounces) filled with 96% ethanol, which were left in place for 72 h. Manual collection was conducted for 1 hectare along each transect. All specimens were preserved in vials containing 96–100% ethanol for subsequent DNA analyses. Sampling was conducted under Research Permit No. 1293 issued to the Universidad del Magdalena.

### Specimen identification

*Crematogaster* species are composed of monomorphic workers, but sometimes vary widely in size (Blaimer 2012a). In most cases, the largest workers with the best morphological integrity were selected for dry mounting using the double mounting technique with acid-free paper point and pin (Guerrero et al. 2019). Identification followed the taxonomic keys of Longino (2003) and Pedraza and Fernández (2019), supplemented with high-resolution species images available on AntWeb (2024) for comparison with the collected *Crematogaster*. A morphospecies was recognized, based on morphological discontinuities observed in comparison with other *Crematogaster* species known from the Neotropical Region. Although this morphospecies is not formally named in the present work, it was included in the numerical analyses and information on its distinctive features is provided.

### Specimen imaging, community composition analyses and distribution maps

**Specimen imaging**

Images of *Crematogaster* workers were obtained using a Leica M205A Auto-Montage microscope, equipped with a DFC450 camera and LAS software v4.6. The specimens imaged are deposited in the CBUMAG collection (see Repositories for definition of the acronym), with individual catalogue numbers listed as CBUMAGENT. All images have been uploaded to AntWeb and are available via the link provided under each species.

**Community composition analyses**

*Sampling unit definition and data preparation*

Ant community composition was analyzed using presence–absence data compiled from three distinct types of data sources: STD, COL and OTH. Given the intrinsic differences in sampling design, effort and detection probability amongst these sources, sampling units were defined as the intersection of site × data source, rather than pooling records across resources. For each site–source combination, a species was scored as present (1) if it was recorded at least once and absent (0) otherwise. This procedure yielded a binary community matrix in which rows correspond to sampling units and columns correspond to ant species. Prior to analysis, species names were taxonomically standardized across datasets to ensure consistency and to avoid artefacts arising from synonymy or inconsistent morphospecies usage.

*Non-metric multidimensional scaling (NMDS)*

Patterns of sampling unit composition were explored using non-metric multidimensional scaling (NMDS), based on a Jaccard dissimilarity matrix, which is appropriate for binary presence–absence data and minimizes the influence of unequal sampling effort amongst sampling units. Ordinations were conducted in two dimensions (k = 2), using multiple random starts to reduce the likelihood of converging on local minima. Ordination quality was evaluated using stress value, with stress < 0.2 considered indicative of an adequate representation of the multivariate dissimilarity structure. NMDS results were visualized with sampling units symbolized according to data source, allowing qualitative assessment of similarities and differences in community composition derived from STD, COL and OTH. NMDS analyses were implemented using the function metaMDS in the R v.4.5.2 packages vegan (R Core Team 2025).

*Permutational multivariate analysis of variance (PERMANOVA)*

To formally test the differences in the composition of the sampling units amongst data sources, we conducted a permutational multivariate analysis of variance (PERMANOVA) using the same Jaccard dissimilarity matrix employed for the NMDS. Data source was treated as a fixed factor. When multiple sampling units originated from the same site, permutations were constrained within sites to account for the lack of independence amongst site-level observations. Statistical significance was assessed using 9,999 permutations. To determine whether significant PERMANOVA results reflected true differences in community composition rather than unequal multivariate dispersion amongst groups, we first tested the homogeneity of dispersions using the function betadisper, followed by a permutation test (ANOVA: *F*₄,₇₃ = 5.26, *p* = 0.0009; permutation test, 999 permutations, *p* = 0.002) (Suppl. material 1). All multivariate analyses were conducted in R v.4.5.2 packages vegan (R Core Team 2025).

**Distribution maps**

Distribution maps of all *Crematogaster* species recorded in the Colombian TDF were produced using R v.4.5.2 (R Core Team 2025). Occurrence data were compiled in an Excel spreadsheet from specimen collection records, including geographic coordinates (latitude and longitude) obtained from specimen labels and imported into R as spatial objects. Spatial data processing and visualization were conducted using the packages sf (Pebesma 2018), sp (Bivand et al. 2013), raster (Hijmans 2010) and ggplot2 (Wickham 2016). The TDF extent was defined using the official ecosystem shapefile provided by the Alexander von Humboldt Biological Resources Research Institute, with departmental boundaries included as base layers. All layers were displayed in the WGS 84 coordinate system (EPSG:4326).

### Repositories

We examined specimens of *Crematogaster* deposited in the following collections:

- **CBUMAG** Centro de Colecciones Biológicas de la Universidad del Magdalena, Santa Marta, Magdalena, Colombia;
- **IAvH** Colección de Entomología del Instituto de Investigaciones de recursos biológicos Alexander von Humboldt, Villa de Leyva, Boyacá, Colombia;
- **ICN** Colección Nacional de Insectos, Instituto de Ciencias Naturales, Universidad Nacional de Colombia, Bogotá, Colombia;
- **MEFLG** Museo Entomológico Francisco Luis Gallego, Universidad Nacional, Medellín, Antioquia, Colombia;
- **MHN UNICAUCA** Museo de Historia Natural Universidad del Cauca, Universidad del Cauca, Popayán, Cauca, Colombia;
- **MUSENUV** Museo de Entomología de la Universidad del Valle, Santiago de Cali, Valle del Cauca, Colombia.

## Taxon treatments

### Crematogaster abstinens

Forel, 1899

32EA021B-3642-510E-8E77-3492A010670C

https://www.antweb.org/specimen/CBUMAGENT41953

#### Distribution

Antioquia, Boyacá, Casanare, Chocó, Guaviare, Huila, Meta, Santander and Vichada (Pedraza and Fernández 2019). In this study, its distribution is extended to the Departments of Atlántico, Cesar, La Guajira, Magdalena, Tolima and Valle del Cauca.

##### Distribution in the TDF

Caribbean Region, Cauca River Valley and Magdalena River Valley.

#### Biology

**Figure 1.**
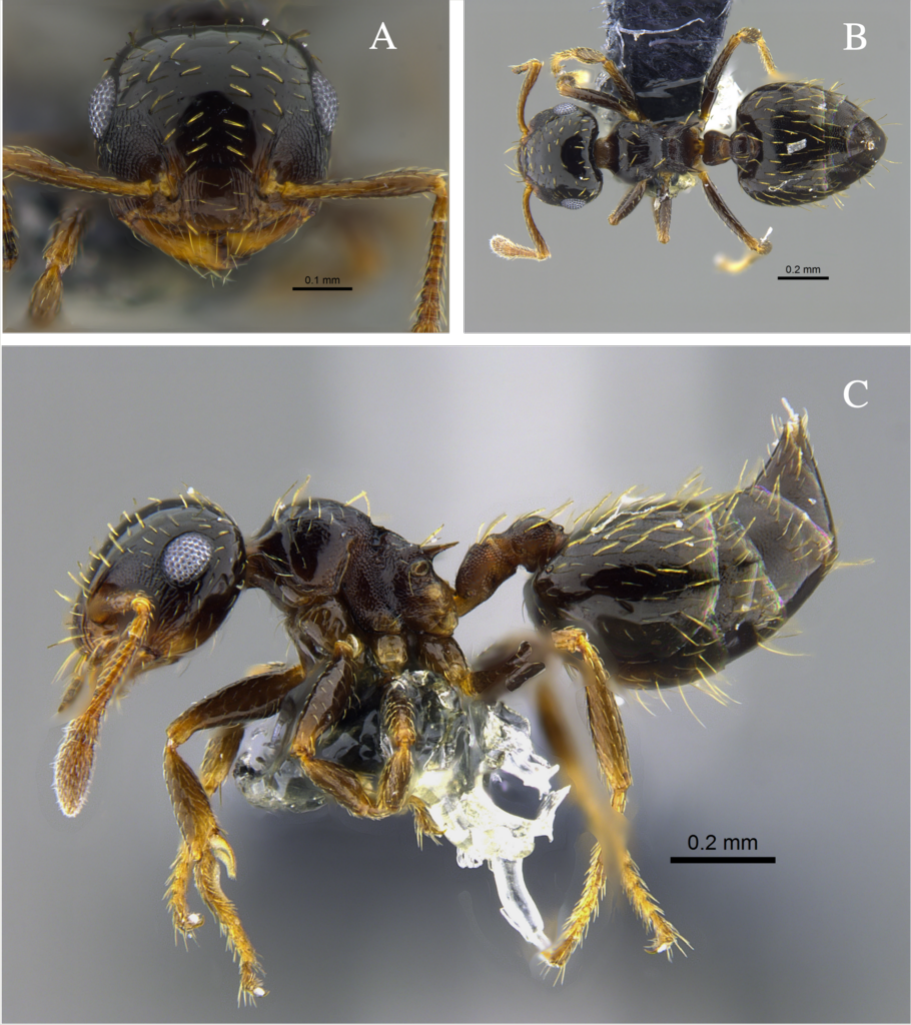
*Crematogaster abstinens* worker (CBUMAGENT41953). **A** full-face view; **B** dorsal view; **C** lateral view.

*Crematogaster abstinens* (Fig. 1) nests mainly in vegetation, but workers were also observed foraging in the leaf litter and, in the Cauca River Valley, at subterranean protein baits.

### Crematogaster ampla

Forel, 1912

9016FAFC-C2B5-5948-9B55-D05286B99290

https://www.antweb.org/specimen/CBUMAGENT41932

#### Distribution

Huila, Magdalena and Valle del Cauca (Pedraza and Fernández 2019). This species extends its known distribution to Antioquia, Bolívar, La Guajira, Santander and Sucre.

##### Distribution in the TDF

Caribbean Region, Cauca River Valley and Magdalena River Valley.

#### Biology

**Figure 2.**
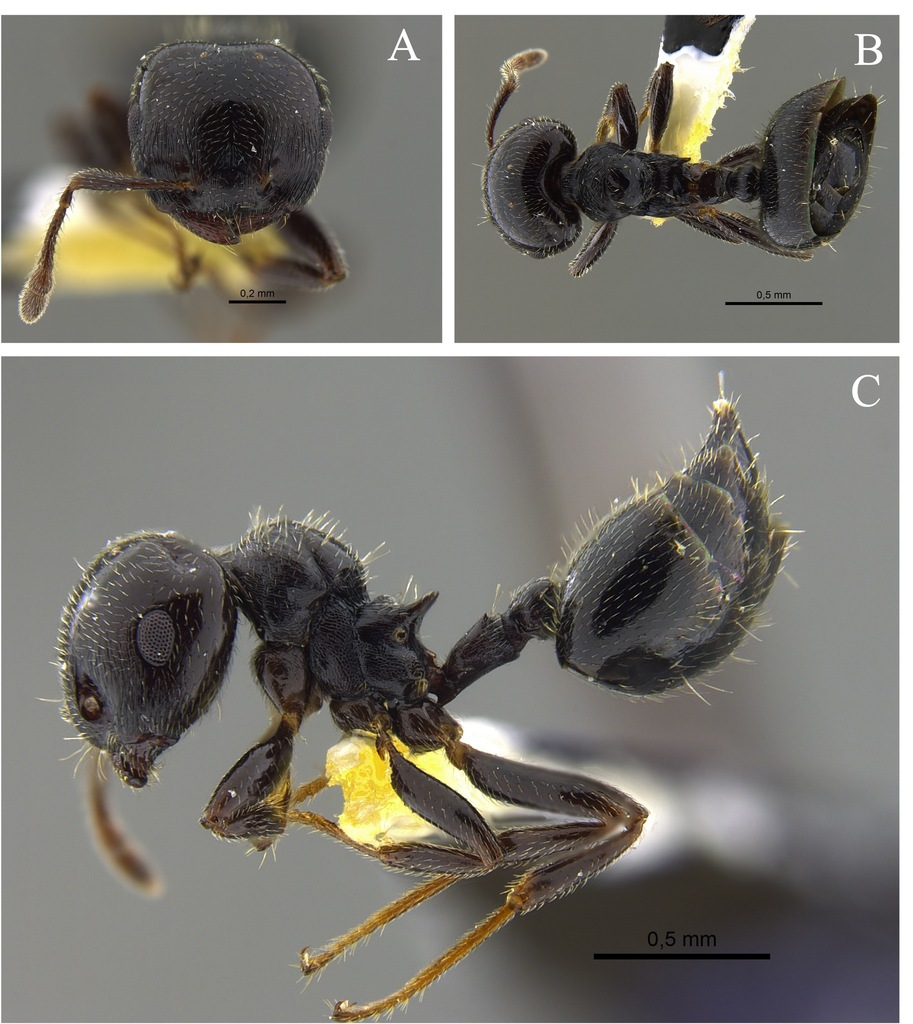
*Crematogaster ampla* worker (CBUMAGENT46667). **A** full-face view; **B** dorsal view; **C** lateral view.

*Crematogaster ampla* (Fig. 2) was found nesting beneath tree bark, attracted to protein baits and foraging in the leaf litter of TDF fragments in the Caribbean and Cauca River Valley regions.

### Crematogaster brasiliensis

Mayr, 1878

54D683E8-1633-5F5D-8B4E-CB9D14A9F3DD

https://www.antweb.org/specimen/CBUMAGENT41935

#### Distribution

Amazonas, Boyacá, Chocó, Guaviare, Magdalena, Meta, Putumayo, Santander and Vaupés (Pedraza and Fernández 2019). This species extends its known distribution to Atlántico, Bolívar, Cesar, La Guajira, Sucre and Valle del Cauca.

##### Distribution in the TDF

Caribbean Region and Cauca River Valley.

#### Biology

**Figure 3.**
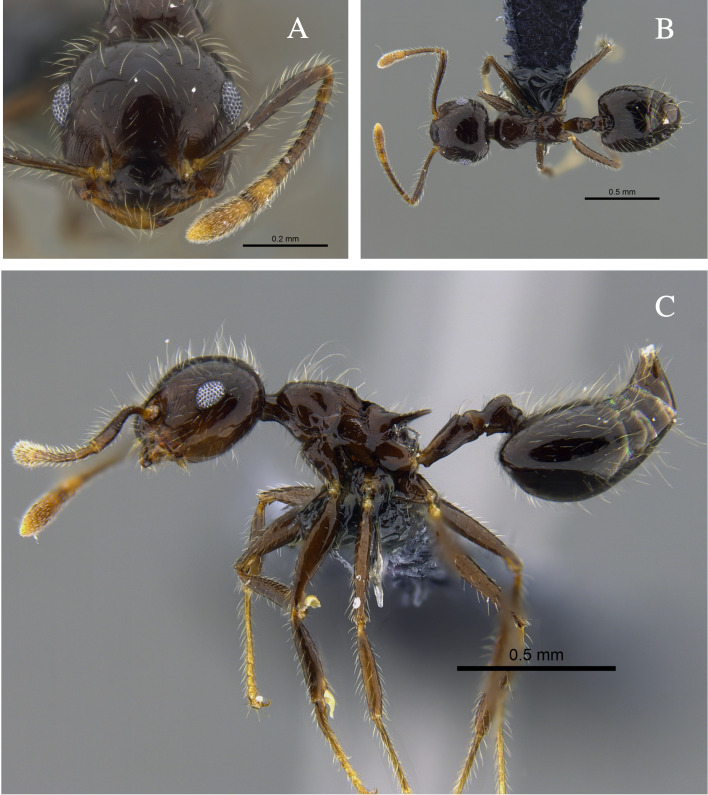
*Crematogaster brasiliensis* worker (CBUMAGENT41935). **A** full-face view; **B** dorsal view; **C** lateral view.

*Crematogaster brasiliensis* (Fig. 3) was found nesting in trunks and dry branches in the localities with TDF from the Caribbean Region, and foraging on tree bark. In remnants of the Cauca River Valley, workers were attracted to protein baits.

### Crematogaster carinata

Mayr, 1862

F7759EF0-596B-596A-8059-91AC8D7ABF33

https://www.antweb.org/specimen/CBUMAGENT41938

#### Distribution

Arauca, Bolívar, Boyacá, Córdoba, Chocó, Huila, Magdalena, Meta, Santander, Sucre, Valle del Cauca and Vaupés (Pedraza and Fernández 2019). This species extends its distribution to Cesar, Sucre, La Guajira and Casanare.

##### Distribution in the TDF

Caribbean Region, Cauca River Valley, Orinoquía and Magdalena River Valley.

#### Biology

**Figure 4.**
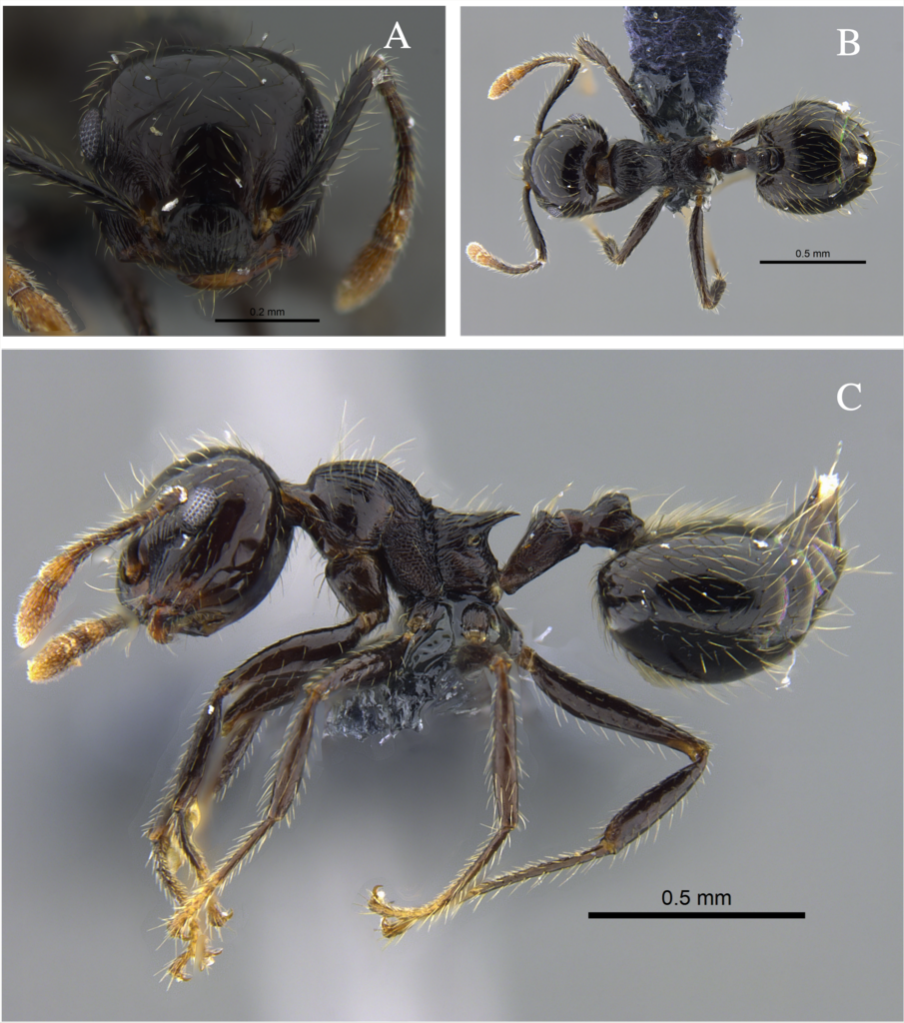
*Crematogaster carinata* worker (CBUMAGENT41938). **A** full-face view; **B** dorsal view; **C** lateral view.

*Crematogaster carinata* (Fig. 4) was found nesting in dry branches and living trees within TDF fragments of the Caribbean Region. The branches contained chambers with larvae, workers and queens. Workers were also collected foraging on vegetation and in the leaf litter. In the Orinoquía fragments, the species was observed foraging both on vegetation and in the leaf litter and, in the Cauca River Valley, it was attracted to protein baits placed on the ground.

### Crematogaster crinosa

Mayr, 1862

CEE02B79-28F5-5FF2-B9A9-1D8B394FEF18

https://www.antweb.org/specimen/CBUMAGENT41951

#### Distribution

Archipiélago de San Andrés y Providencia, Antioquia, Bolívar, Boyacá, Cesar, Cundinamarca, Chocó, La Guajira, Huila, Magdalena (Type locality), Meta and Santander (Pedraza and Fernández 2019). This species extends its distribution to Sucre and Valle del Cauca.

##### Distribution in the TDF

Caribbean Region, Cauca River Valley and Magdalena River Valley.

#### Biology

**Figure 5.**
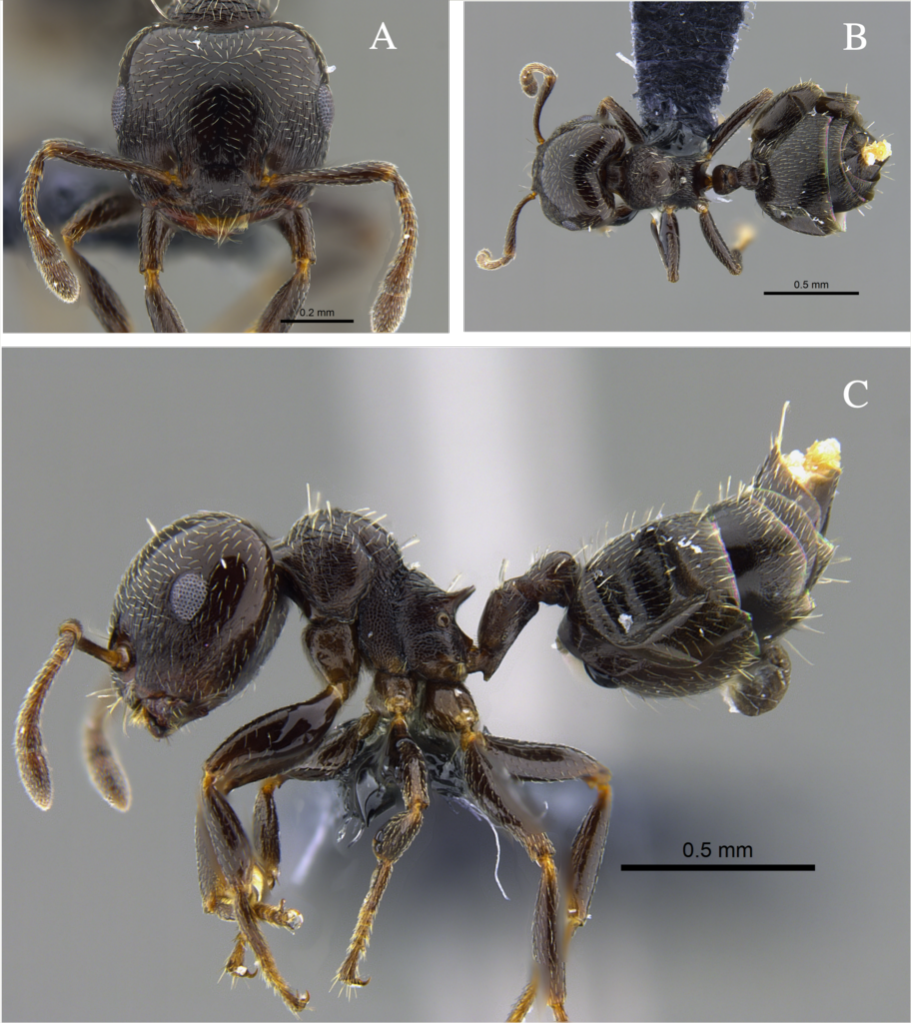
*Crematogaster crinosa* worker (CBUMAGENT41951). **A** full-face view; **B** dorsal view; **C** lateral view.

*Crematogaster crinosa* (Fig. 5) was observed foraging both on vegetation and in the leaf litter. In TDF fragments of the Cauca River Valley Region, workers were collected using protein baits, whereas in the Caribbean Region, the species was found nesting beneath the bark of a hard dead trunk and within a carton nest built on a live branch.

### Crematogaster crucis

Forel, 1912

1978C8E4-B5BE-551E-B273-D5E3F4DA5646

https://www.antweb.org/specimen/CBUMAGENT41933

#### Distribution

Chocó, César and Magdalena (Pedraza and Fernández 2019).

##### Distribution in the TDF

Widely distributed in the Colombian Caribbean Region.

#### Biology

**Figure 6.**
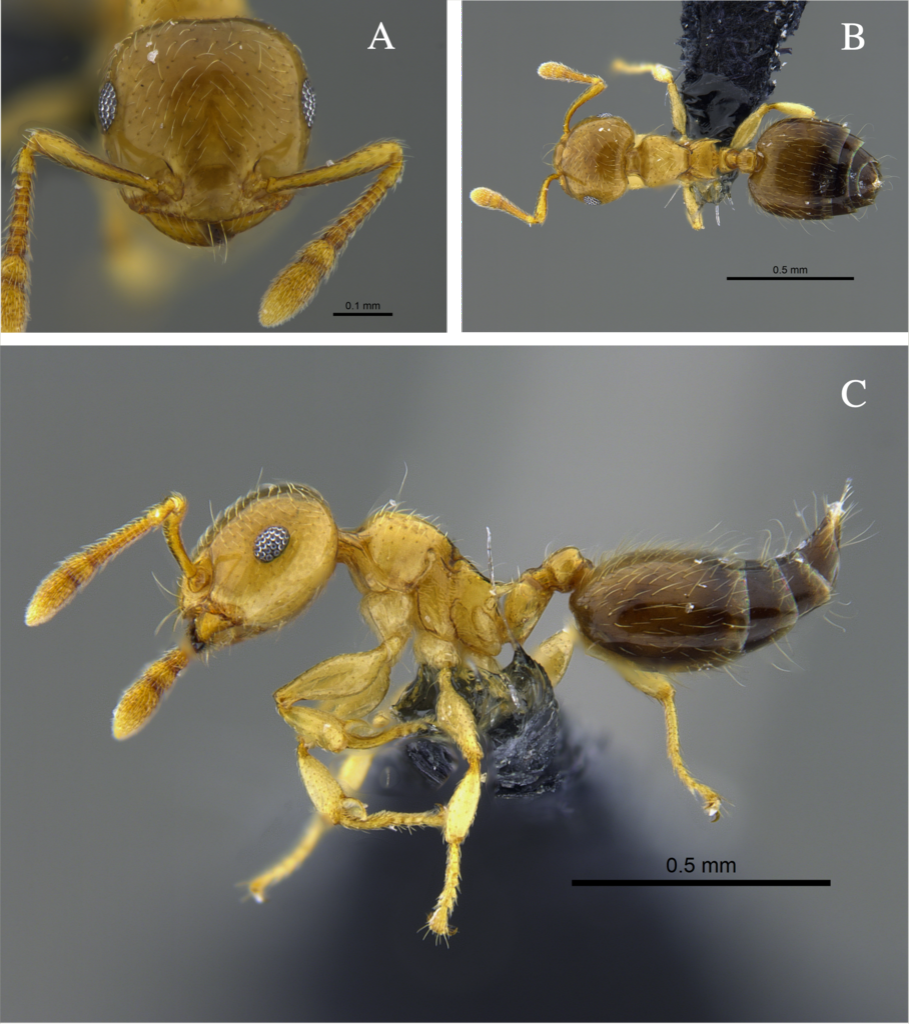
*Crematogaster crucis* worker (CBUMAGENT41933). **A** full-face view; **B** dorsal view; **C** lateral view.

*Crematogaster crucis* (Fig. 6) was observed foraging in vegetation, leaf litter and open ground in TDF fragments of the Caribbean Region; it was collected exclusively by manual sampling. Some records were obtained from dry branches of *Coccoloba* (Polygonaceae) located at the edge between the beach and the tropical dry forest in Tayrona National Park (Longino 2003).

### Crematogaster curvispinosa

Mayr, 1862

DD45C031-C1BE-5DA9-A5C5-896318562D31

https://www.antweb.org/specimen/CBUMAGENT41942

#### Distribution

Antioquia, Bolívar, Boyacá, Cauca, Cundinamarca, Huila, Magdalena, Meta, Nariño, Risaralda, Santander and Valle del Cauca (Pedraza and Fernández 2019). This species extends its distribution to Arauca, Atlántico, Bolívar, Cesar, La Guajira and Tolima.

##### Distribution in the TDF

Caribbean Region, Cauca River Valley, Patía River Valley, Orinoquia and Magdalena River Valley.

#### Biology

**Figure 7.**
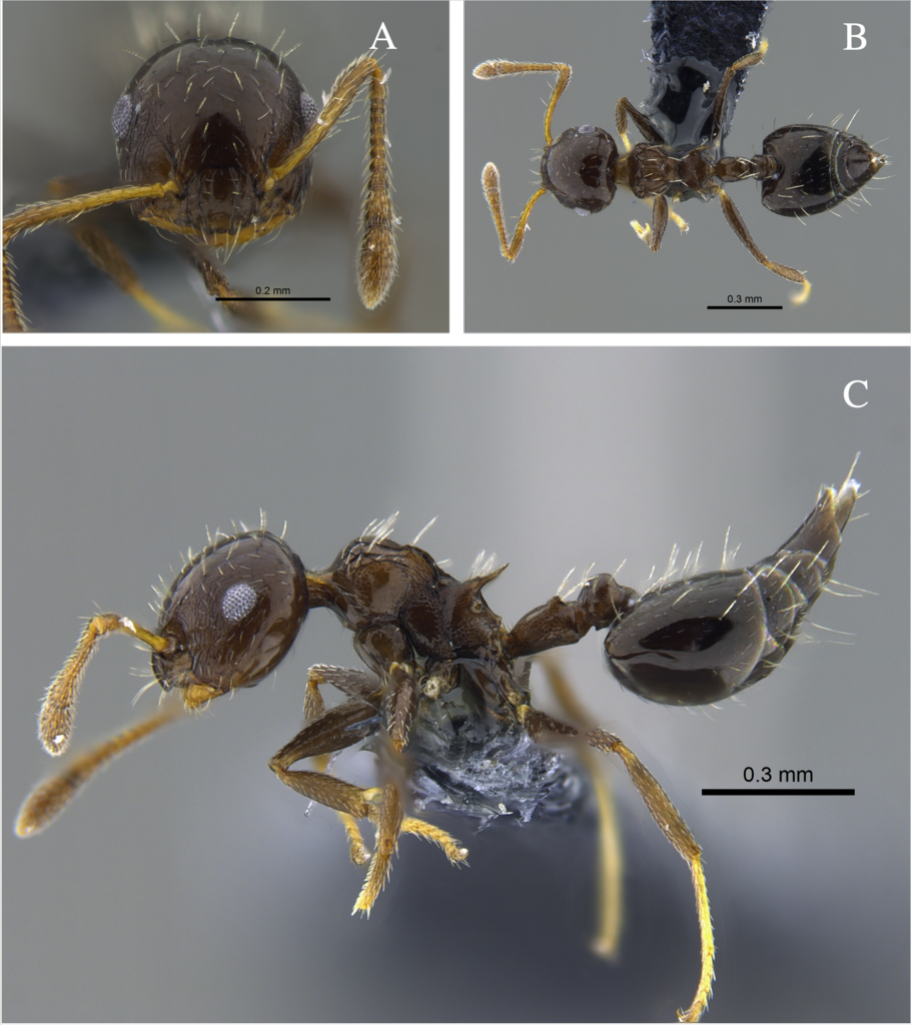
*Crematogaster curvispinosa* worker (CBUMAGENT41942). **A** full-face view; **B** dorsal view; **C** lateral view.

*Crematogaster curvispinosa* (Fig. 7) was observed nesting and foraging on the bark of *Mangifera indica* and foraging on the trunk of *Ceratonia europaea*, both tree species being present in TDF fragments of the Caribbean Region. Workers were also recorded foraging inside the flowers of an unidentified climbing plant. In forest fragments of the Cauca River Valley Region, the species was attracted to protein baits placed on the leaf litter.

### Crematogaster distans

Mayr, 1870

D7F50FD3-9AD5-5E73-8751-E8E6F2773863

https://www.antweb.org/specimen/IAVH-E-262542

#### Distribution

Antioquia, Boyacá, Cundinamarca, Huila, Magdalena and Santander (Pedraza and Fernández 2019). This species extends its distribution to Córdoba and Tolima.

##### Distribution in the TDF

Caribbean Region and Magdalena River Valley.

#### Biology

**Figure 8.**
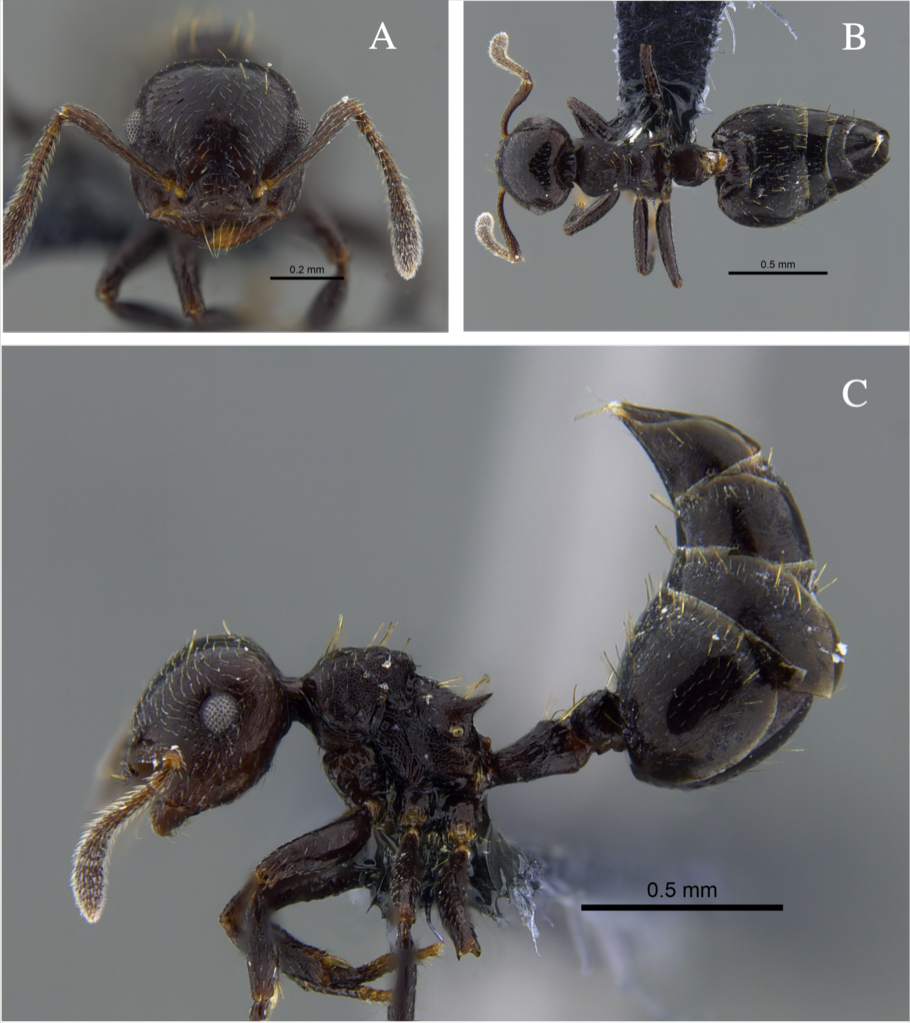
*Crematogaster distans* worker (IAVH-E-262542). **A** full-face view; **B** dorsal view; **C** lateral view.

*Crematogaster distans* (Fig. 8) was observed foraging in the leaf litter in the Magdalena River Valley Region, whereas in the Caribbean Region, workers were recorded foraging both on vegetation and in the litter.

### Crematogaster erecta

Mayr, 1866

42D6D96C-8104-5684-B621-B0372368AA73

https://www.antweb.org/specimen/CBUMAGENT41941

#### Distribution

Boyacá, Cesar, Cundinamarca, Chocó, Magdalena, Meta, Nariño, Santander and Valle del Cauca (Pedraza and Fernández 2019). This species extends its distribution to the Archipiélago de San Andrés y Providencia, Atlántico, Bolívar, Cauca and Huila.

##### Distribution in the TDF

Caribbean Region, Cauca River Valley, Patía River Valley and Magdalena River Valley.

#### Biology

**Figure 9.**
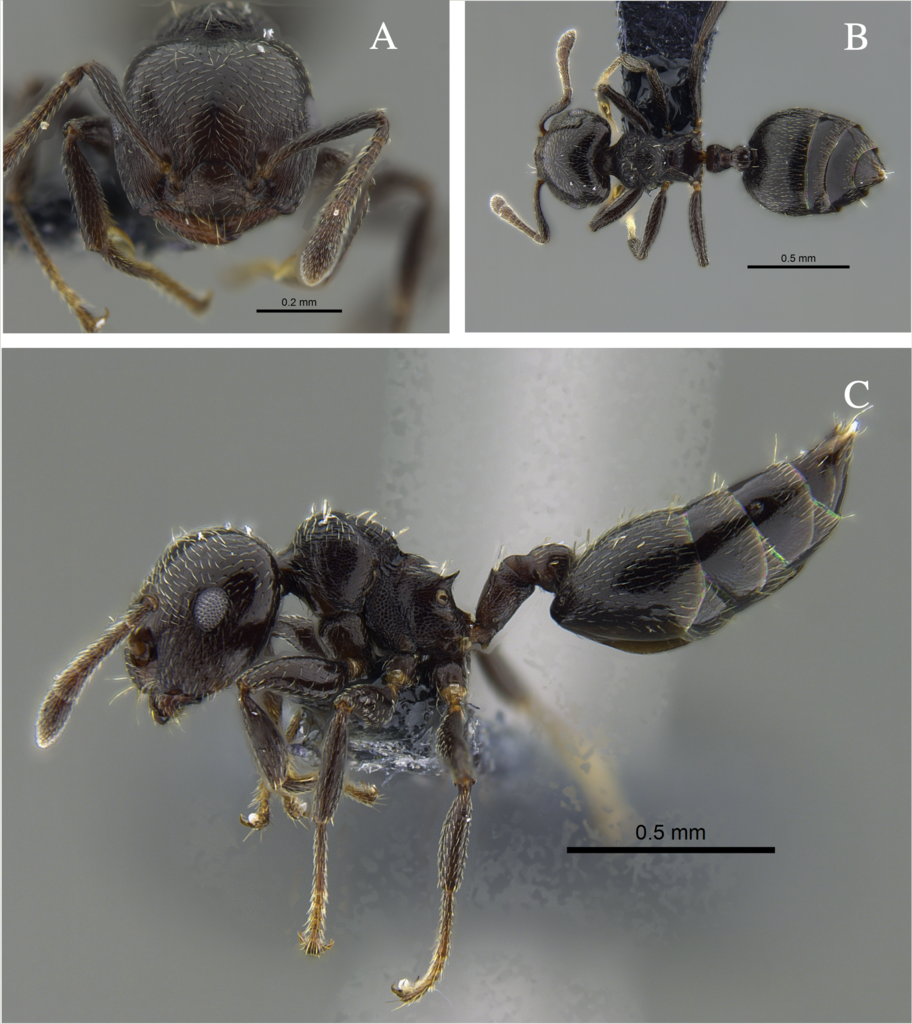
*Crematogaster erecta* worker (CBUMAGENT41941). **A** full-face view; **B** dorsal view; **C** lateral view.

*Crematogaster erecta* (Fig. 9) was observed foraging on vegetation and in the leaf litter of TDF fragments in the Caribbean Region. In the fragments of the Cauca River Valley, Patía River Valley and Magdalena River Valley regions, it was found exclusively associated with the leaf litter.

### Crematogaster evallans

Forel, 1907

92B1558F-DB4B-5A45-AB3E-769AF39AA779

https://www.antweb.org/specimen/CBUMAGENT41937

#### Distribution

Chocó, Huila, Magdalena, Meta, Santander and Valle del Cauca (Pedraza and Fernández 2019). This species extends its distribution to Antioquia.

##### Distribution in the TDF

Cauca River Valley and Magdalena River Valley.

#### Biology

**Figure 10.**
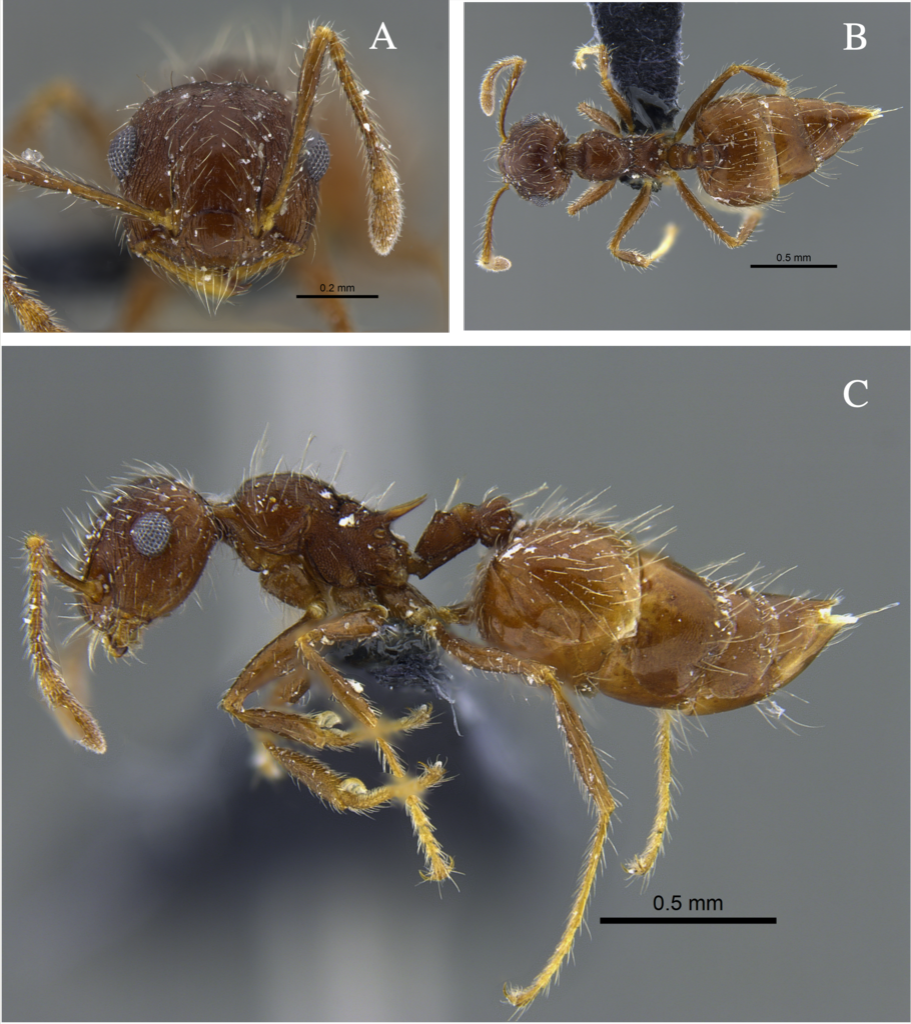
*Crematogaster evallans* worker (CBUMAGENT41937). **A** full-face view; **B** dorsal view; **C** lateral view.

*Crematogaster evallans* (Fig. 10) was observed foraging in the leaf litter of forest fragments in the Magdalena River Valley. In the fragments of the Cauca River Valley, it was found foraging in *Mangifera indica* plantations and was attracted to both protein and carbohydrate baits.

### Crematogaster flavosensitiva

Longino, 2003

26B0232E-8266-5B20-8221-3A58AC67B9DB

https://www.antweb.org/specimen/CBUMAGENT41955

#### Distribution

Amazonas and Magdalena (Pedraza and Fernández 2019). This species extends its distribution to Arauca, Atlántico, Bolívar, Casanare, Cesar, La Guajira and Sucre.

##### Distribution in the TDF

Caribbean Region and Orinoquía Region.

#### Biology

**Figure 11.**
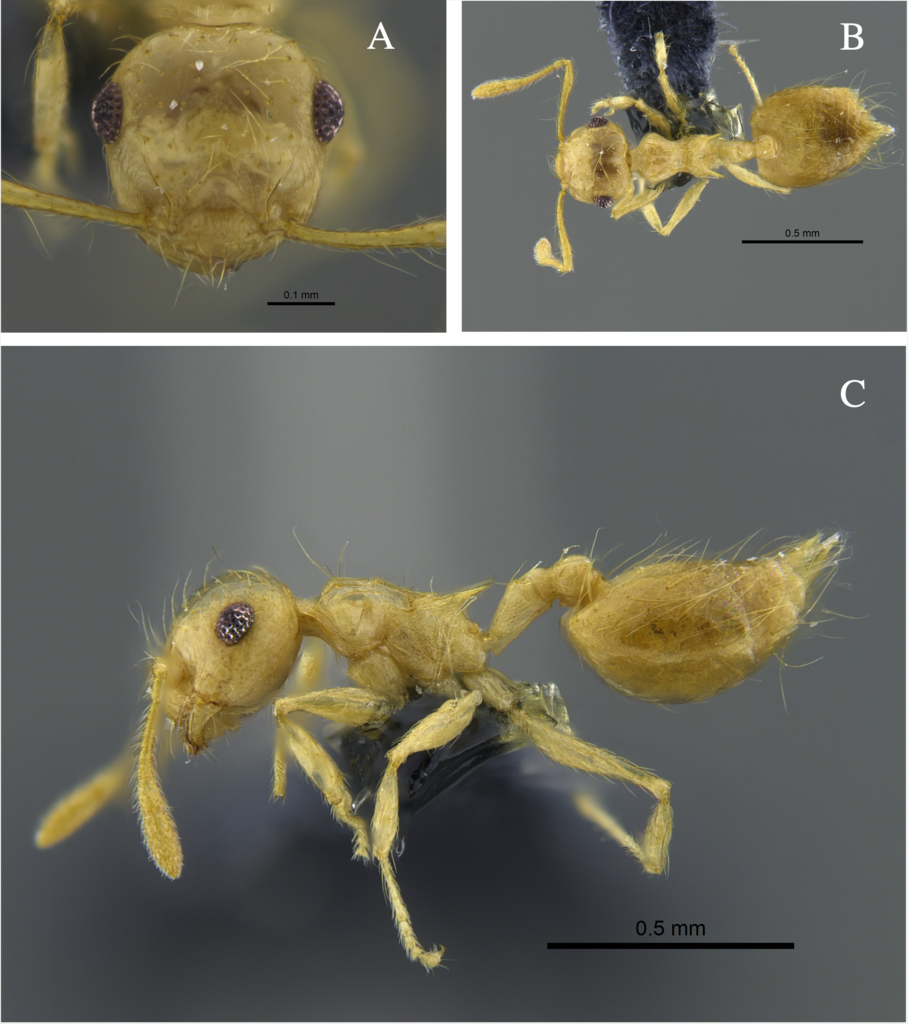
*Crematogaster flavosensitiva* worker (CBUMAGENT41955). **A** full-face view; **B** dorsal view; **C** lateral view.

*Crematogaster flavosensitiva* (Fig. 11) was observed foraging on vegetation and in the leaf litter in forest fragments located in the Caribbean Region; in the Orinoquía, it was collected only from the leaf litter.

### Crematogaster goeldii

Forel, 1903

E7D97DA9-28D5-51DF-82B9-C9E4E89E4603

https://www.antweb.org/specimen/CBUMAGENT41930

#### Distribution

This species was previously known from Argentina, Bolivia, Brazil and Guyana. This species extends its distribution to Colombia, specifically in the Department of Atlántico.

##### Distribution in the TDF

Caribbean Region.

#### Biology

**Figure 12.**
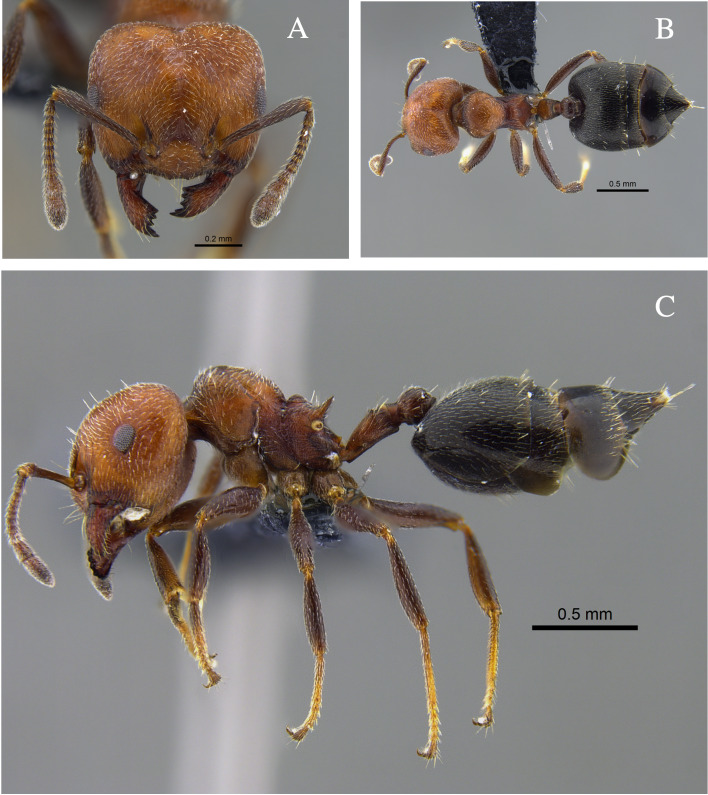
*Crematogaster goeldii* worker (CBUMAGENT41930). **A** full-face view; **B** dorsal view; **C** lateral view.

*Crematogaster goeldii* (Fig. 12) was observed foraging on shrub vegetation during night-time in the studied TDF fragments of the Caribbean Region.

### Crematogaster levior

Longino, 2003

76A85F22-3E43-5D2B-AF0D-737D5C8A4E4E

https://www.antweb.org/specimen/CBUMAGENT41936

#### Distribution

Amazonas, Boyacá, Cundinamarca, Chocó, Meta, Putumayo and Valle del Cauca (Pedraza and Fernández 2019). This species extends its distribution to Atlántico, Bolívar and Tolima.

##### Distribution in the TDF

Caribbean Region and Magdalena River Valley.

#### Biology

**Figure 13.**
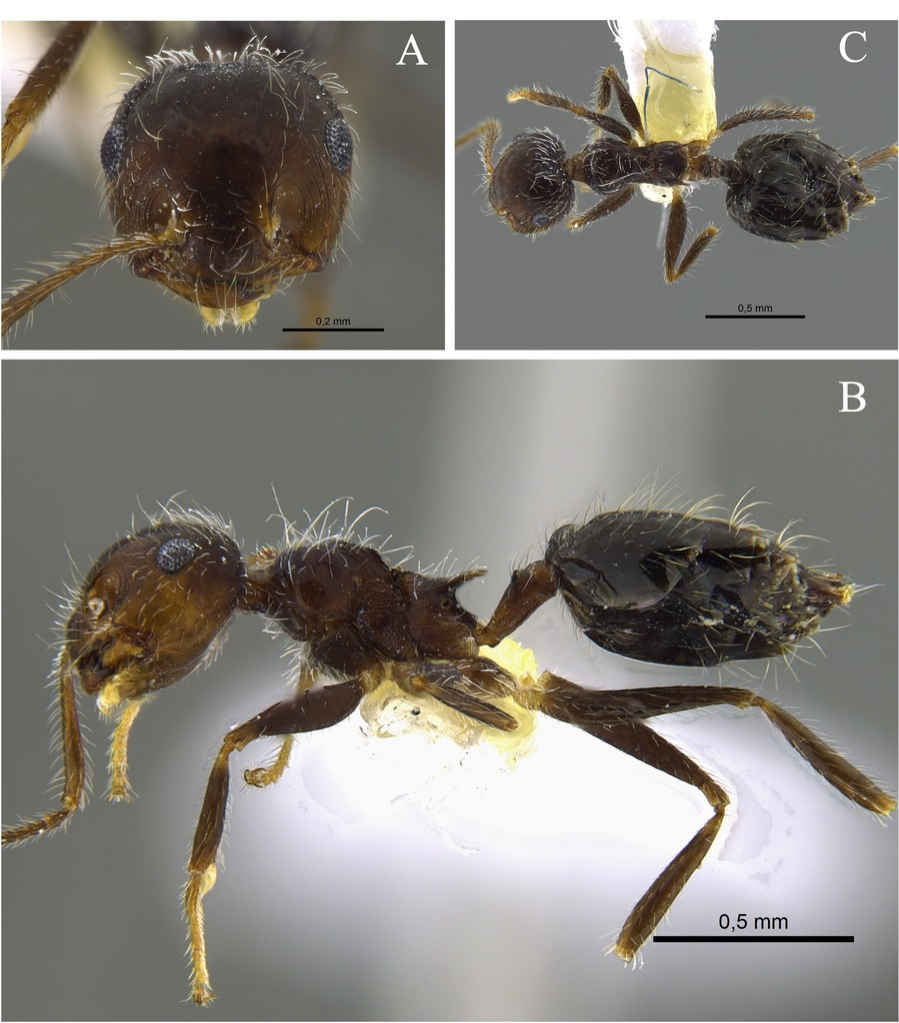
*Crematoagster levior* worker (CBUMAGENT46668). **A** full-face view; **B** dorsal view; **C** lateral view.

*Crematogaster levior* (Fig. 13) was extracted from the leaf litter in TDF fragments of Caribbean and Magdalena River Valley regions. It has been observed nesting in association with *Camponotus femoratus* (Fabricius, 1804).

### Crematogaster limata

Smith, 1858

DA98C790-04A5-5635-BC36-02CA27DDAF82

https://www.antweb.org/specimen/CBUMAGENT41952

#### Distribution

Amazonas, Antioquia, Cundinamarca, Chocó, Guaviare, Huila, Magdalena, Meta, Putumayo, Risaralda, Santander, Tolima, Valle del Cauca and Vichada (Pedraza and Fernández 2019). This species extends its distribution to Arauca, Atlántico, Bolívar, Casanare, Cesar, La Guajira and Sucre.

##### Distribution in the TDF

Caribbean Region, Orinoquía Region, Patía River Valley and Magdalena River Valley.

#### Biology

**Figure 14.**
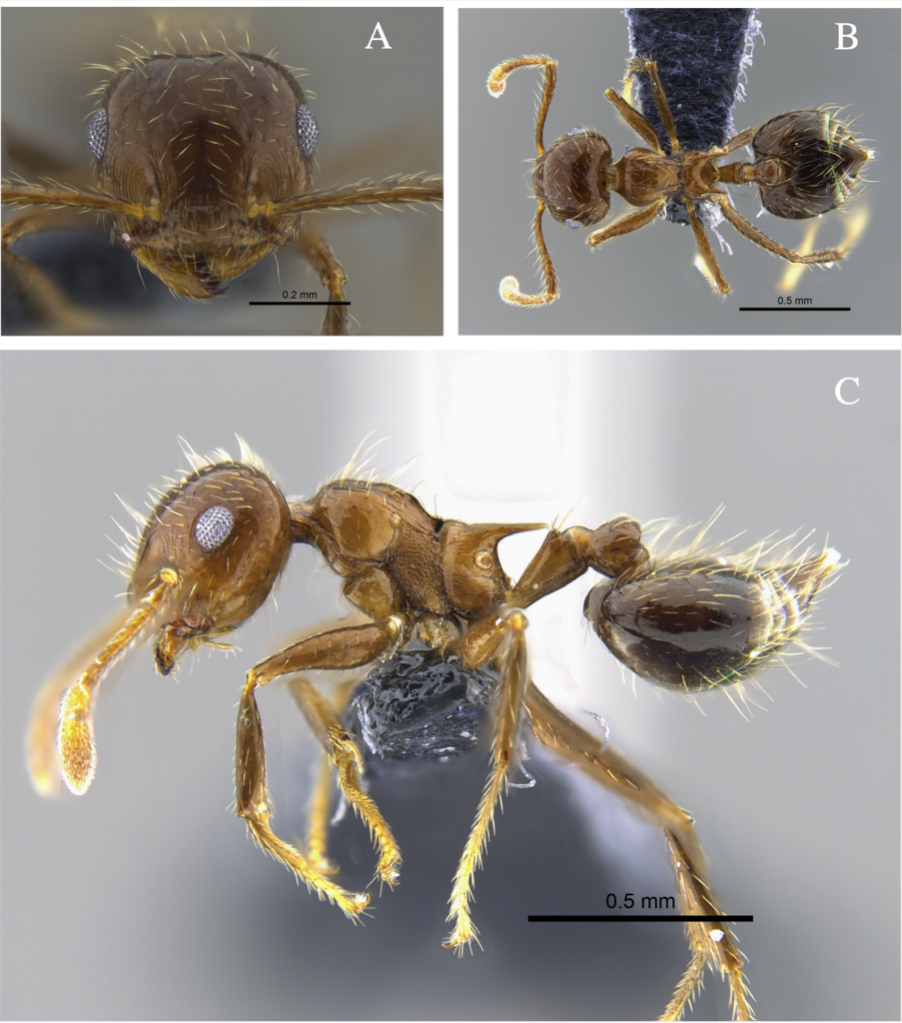
*Crematogaster limata* worker (CBUMAGENT41952). **A** full-face view; **B** dorsal view; **C** lateral view.

*Crematogaster limata* (Fig. 14) was observed in forest fragments of Magdalena (Caribbean Region) nesting beneath the bark of an *Anacardium excelsum* tree. The species was also found nesting under the bark of a decaying log covered with moss and in cavities inside trunks perforated by termites. In addition, individuals were collected from an elongated carton nest containing a polygynous colony with three queens and workers were observed foraging in the leaf litter. In the Orinoquía Region and the Magdalena River Valley, workers were found exclusively in the litter.

### Crematogaster montezumia

Smith, 1858

90C6A387-75EB-52F6-93F0-22728E3B0FF0

https://www.antweb.org/specimen/CBUMAGENT41954

#### Distribution

Magdalena and Meta (Pedraza and Fernández 2019). This species extends its distribution to Cauca, Huila and Valle del Cauca.

##### Distribution in the TDF

Cauca River Valley, Magdalena River Valley and Patía River Valley.

#### Biology

**Figure 15.**
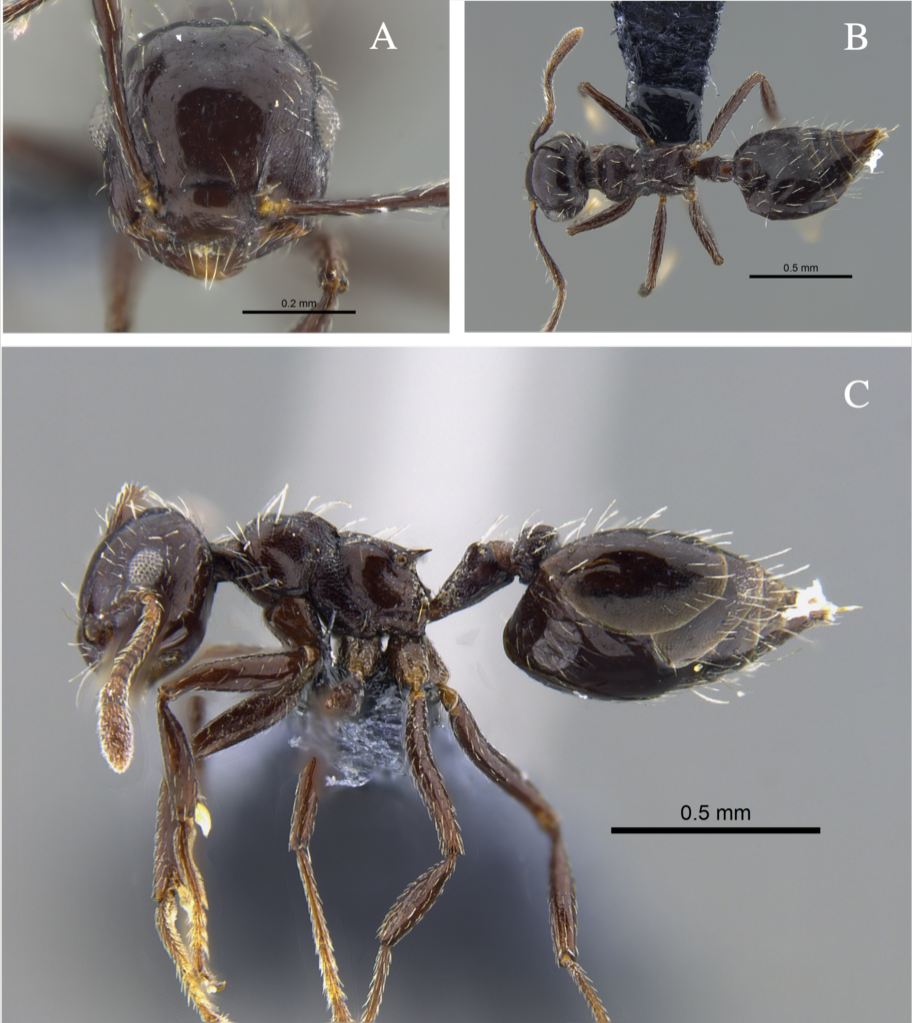
*Crematogaster montezumia* worker (CBUMAGENT41954). **A** full-face view; **B** dorsal view; **C** lateral view.

*Crematogaster montezumia* (Fig. 15) was observed foraging on vegetation in tropical dry forest (TDF) fragments of the Cauca River Valley, Magdalena River Valley and Patía River Valley regions. However, in these regions, it was also found exclusively in the leaf litter and on the ground.

### Crematogaster nigropilosa

Mayr, 1870

A6815A6D-9E1D-5EF8-9DA5-850FBEEC558C

https://www.antweb.org/specimen/CBUMAGENT41940

#### Distribution

Boyacá, Cundinamarca, Chocó, Guaviare, Huila, Magdalena, Meta, Risaralda, Santander, Tolima and Valle del Cauca (Pedraza and Fernández 2019). This species extends its distribution to Atlántico, Bolívar, Casanare, Cesar and La Guajira.

##### Distribution in the TDF

Caribbean Region, Cauca River Valley, Orinoquía and Magdalena River Valley.

#### Biology

**Figure 16.**
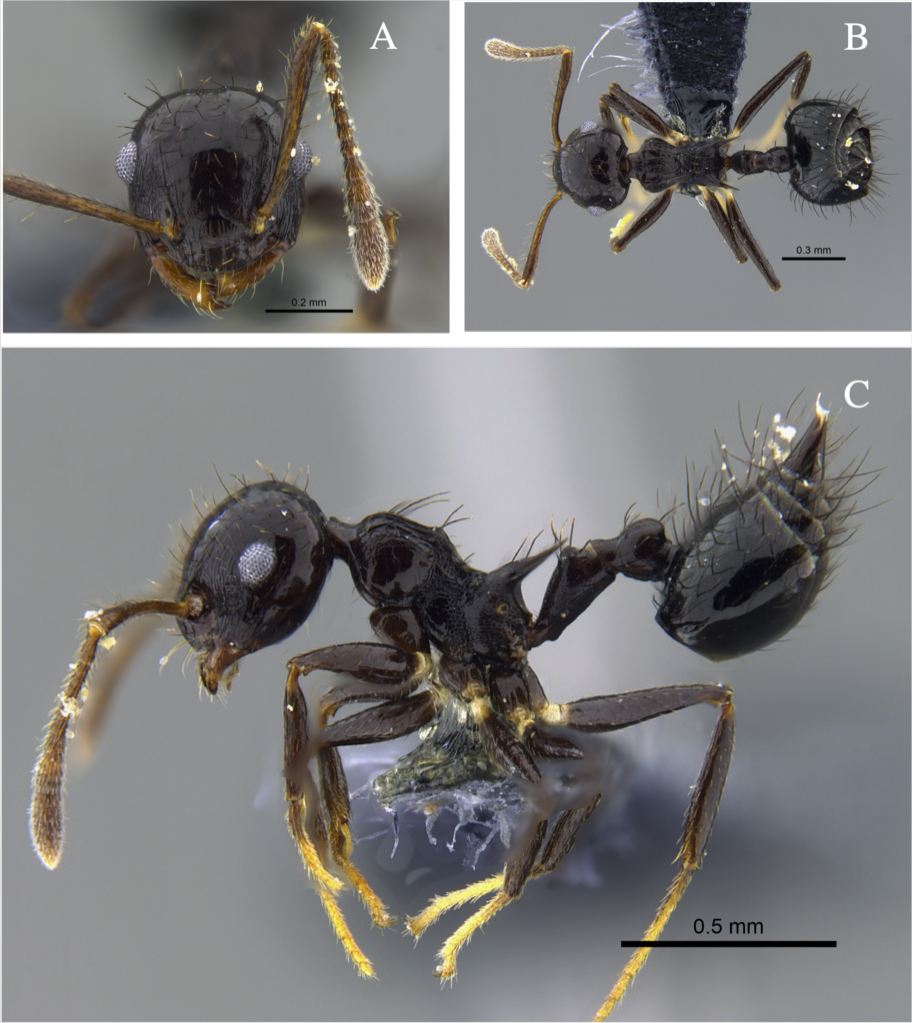
*Crematogaster nigropilosa* worker (CBUMAGENT41940). **A** full-face view; **B** dorsal view; **C** lateral view.

*Crematogaster nigropilosa* (Fig. 16) was observed foraging on vegetation and in the leaf litter of tropical dry forest (TDF) fragments in the Caribbean Region. In other regions, it was found foraging in the litter and on the ground.

### Crematogaster nitidiceps

Emery, 1895

178C374B-7CA8-572B-80BC-D87F2AEB6E0D

https://www.antweb.org/specimen/CBUMAGENT41939

#### Distribution

Magdalena and Meta (Pedraza and Fernández 2019). This species extends its distribution to Córdoba and La Guajira.

##### Distribution in the TDF

Caribbean Region.

#### Biology

**Figure 17.**
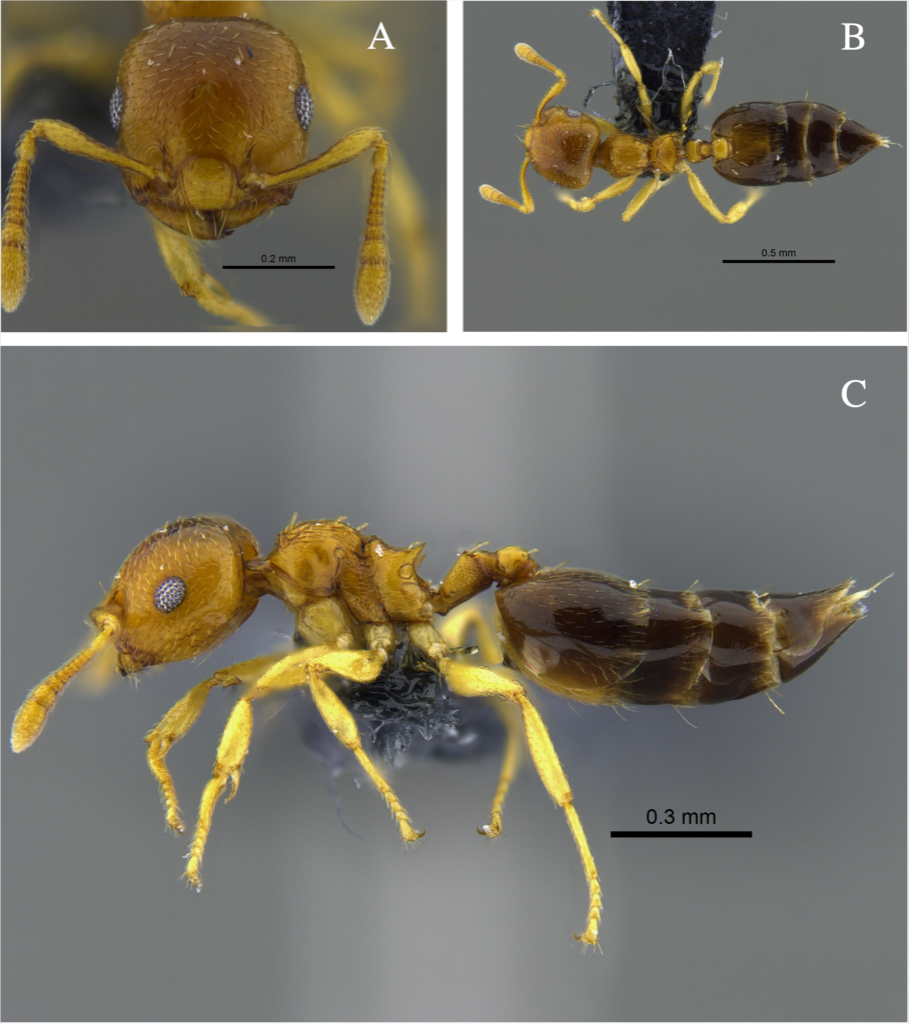
*Crematogaster nitidiceps* worker (CBUMAGENT41939). **A** full-face view; **B** dorsal view; **C** lateral view.

*Crematogaster nitidiceps* (Fig. 17) was observed nesting and foraging primarily on vegetation associated with tropical dry forest (TDF) fragments in the Caribbean Region. Individuals were collected nesting in a decaying log next to a shrub and foraging on the bark of *Quercus robur*, where nests were also found inside an abandoned termite mound. Additionally, nests were located in the palm *Bactris guineensis* and workers were collected from the leaf litter and the ground.

### Crematogaster obscurata

Emery, 1895

953E744A-3A71-54E3-B52D-74120F478EBE

https://www.antweb.org/specimen/CBUMAGENT42077

#### Distribution

Magdalena, Meta and Santander (Pedraza and Fernández 2019). This species extends its distribution to Atlántico and Valle del Cauca.

##### Distribution in the TDF

Caribbean Region and Cauca River Valley.

#### Biology

**Figure 18.**
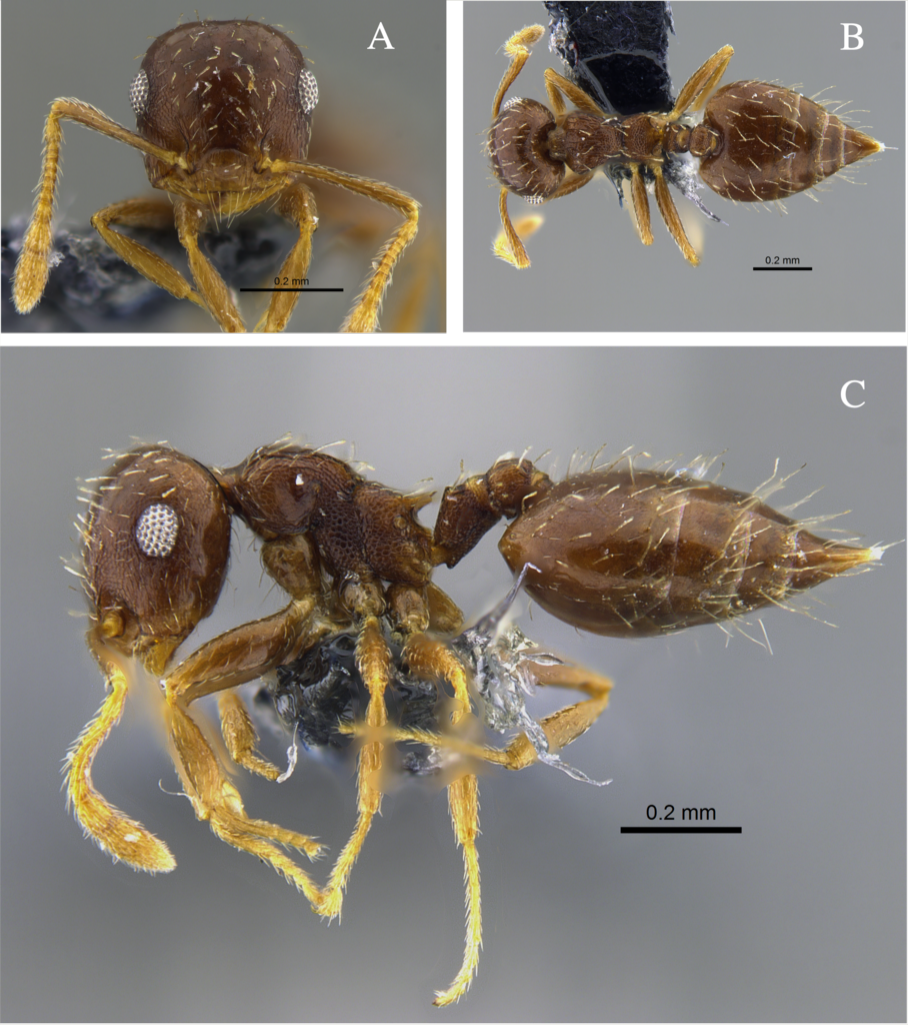
*Crematogaster obscurata* worker (CBUMAGENT42077). **A** full-face view; **B** dorsal view; **C** lateral view.

*Crematogaster obscurata* (Fig. 18) was observed foraging and nesting in vegetation, leaf litter and on the ground in tropical dry forest (TDF) fragments of the Caribbean Region. In contrast, in the Cauca River Valley fragments, it was collected only from vegetation. This species is commonly found in dry forests.

### Crematogaster rochai

Forel, 1903

9112F860-B205-5860-B394-5CEB08FCE538

https://www.antweb.org/specimen/CBUMAGENT41934

#### Distribution

La Guajira, Huila, Magdalena, Meta and Santander (Pedraza and Fernández 2019). This species extends its distribution to Antioquia, Arauca, Atlántico and Cesar.

##### Distribution in the TDF

Caribbean Region, Cauca River Valley, Orinoquía and Magdalena River Valley.

#### Biology

**Figure 19.**
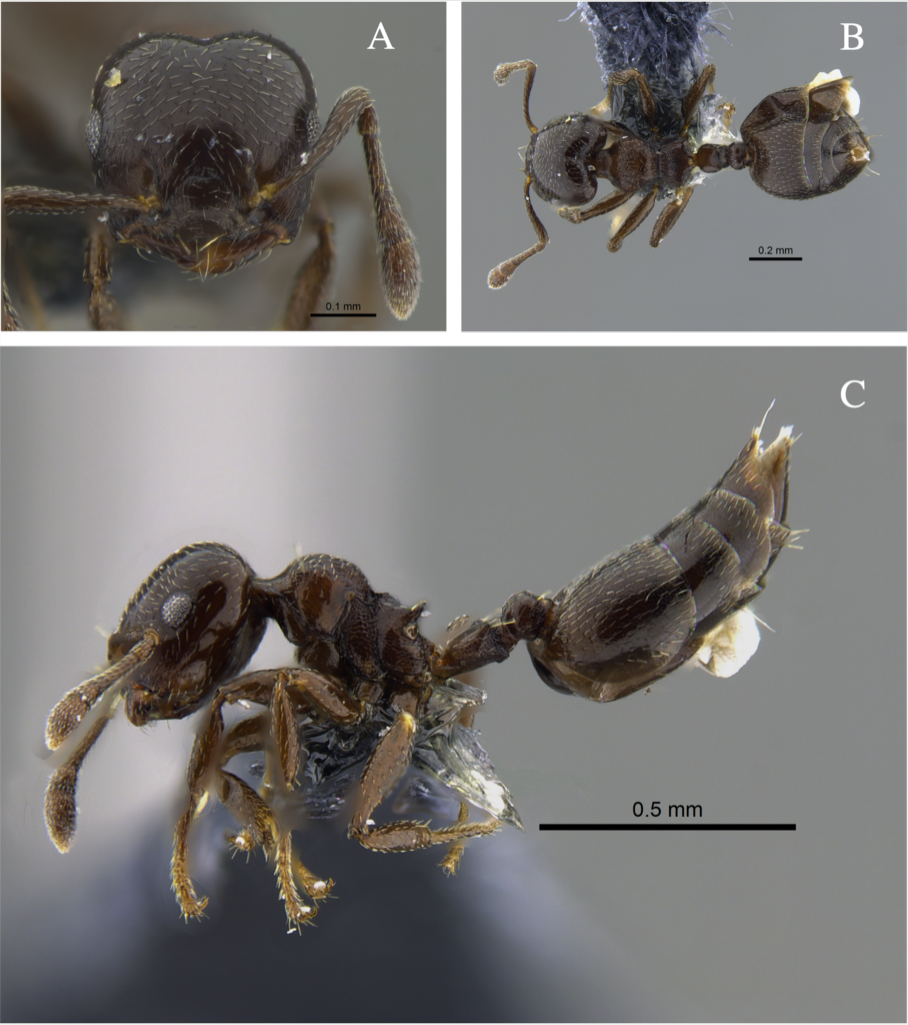
*Crematogaster rochai* worker (CBUMAGENT41934). **A** full-face view; **B** dorsal view; **C** lateral view.

*Crematogaster rochai* (Fig. 19) was found in tropical dry forest (TDF) fragments of the Caribbean Region, foraging on an *Olea europaea* shrub located near the beach. It was also observed nesting in a shrub belonging to the subfamily Mimosoideae, as well as excavating galleries inside live branches and both nesting and foraging on *Mangifera indica*. In fragments of Cauca River Valley and Magdalena River Valley, individuals were collected using protein baits and were also found on the ground, in the leaf litter and on vegetation. Longino (2003) notes that this species is common in disturbed areas.

### Crematogaster sotobosque

Longino, 2003

915222C2-7E38-507A-9244-BF99A15BD68A

https://www.antweb.org/specimen/CBUMAGENT41931

#### Distribution

Amazonas, Boyacá, Cundinamarca, Chocó, Meta, Risaralda and Valle del Cauca (Pedraza and Fernández 2019).

##### Distribution in the TDF

Cauca River Valley Region.

#### Biology

**Figure 20.**
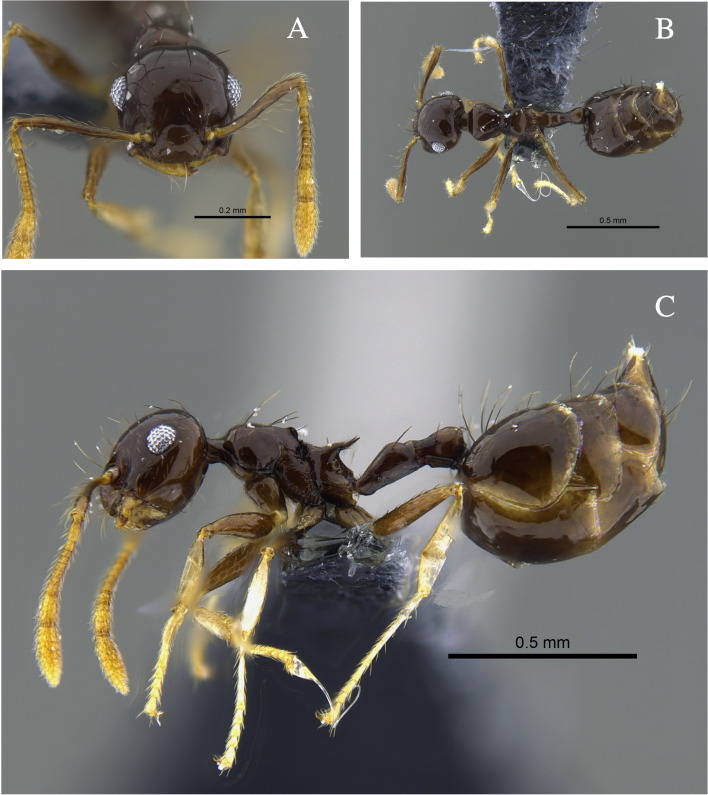
*Crematogaster sotobosque* worker (CBUMAGENT41931). **A** full-face view; **B** dorsal view; **C** lateral view.

*Crematogaster sotobosque* (Fig. 20) was attracted to protein baits placed on the ground and was also observed foraging in the leaf litter of TDF fragments in the Cauca River Valley.

### Crematogaster stollii

Forel, 1885

6AF32B75-63B3-5A9A-B658-ACAEF4B25E59

https://www.antweb.org/specimen/CBUMAGENT41929

#### Distribution

Amazonas, Antioquia, Cauca, Chocó and La Guajira (Pedraza and Fernández 2019). This species extends its distribution to Atlántico and Tolima.

##### Distribution in the TDF

Caribbean Region and Magdalena River Valley regions.

#### Biology

**Figure 21.**
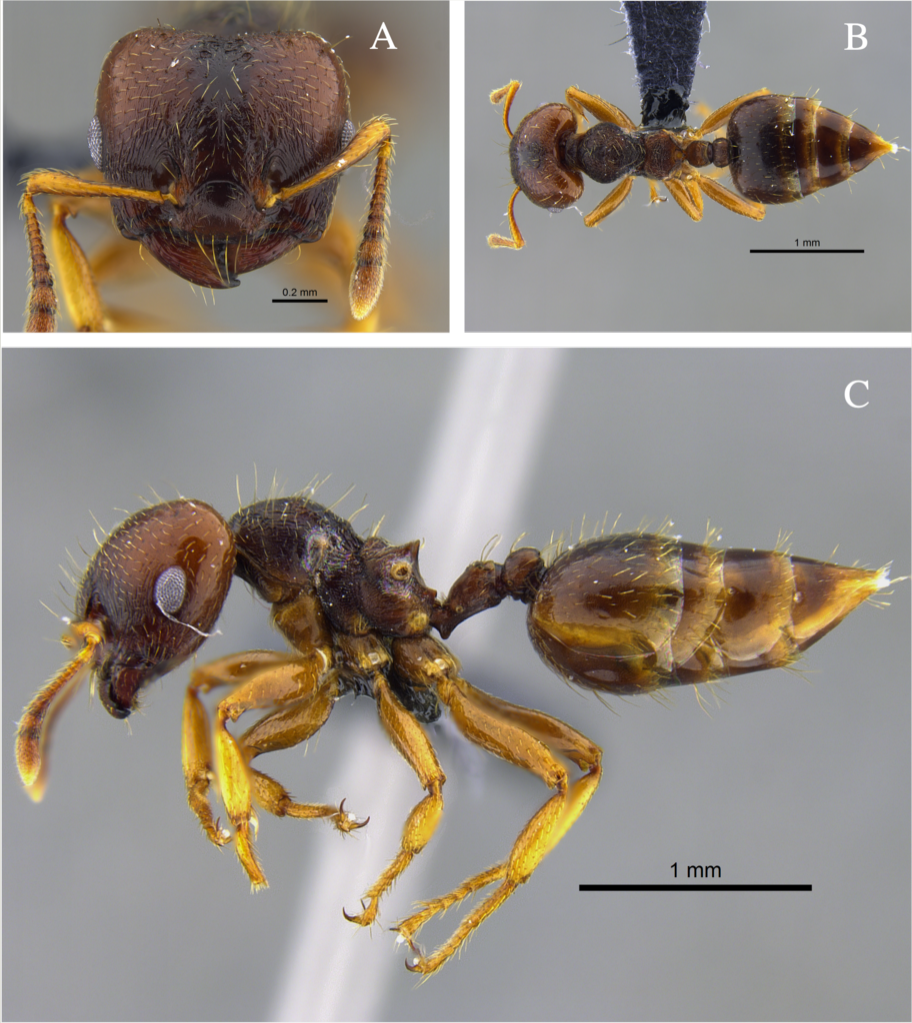
*Crematogaster stollii* worker (CBUMAGENT41929). **A** full-face view; **B** dorsal view; **C** lateral view.

*Crematogaster stollii* (Fig. 21) was observed in dry forest fragments of the Caribbean Region foraging on the bark of *Quercus humboldtii* and inside an abandoned termite nest. In the Magdalena River Valley Region, individuals were collected directly from the ground.

### Crematogaster torosa

Mayr, 1870

D54FAE19-E133-54C8-81B6-EB4AAE53A3B9

https://www.antweb.org/specimen/CBUMAGENT41928

#### Distribution

Huila, Magdalena, Santander and Valle del Cauca (Pedraza and Fernández 2019). This species extends its distribution to Archipiélago de San Andrés y Providencia, Atlántico, Cauca and Tolima.

##### Distribution in the TDF

Caribbean Region, Cauca River Valley, Magdalena River Valley and Patía River Valley.

#### Biology

**Figure 22.**
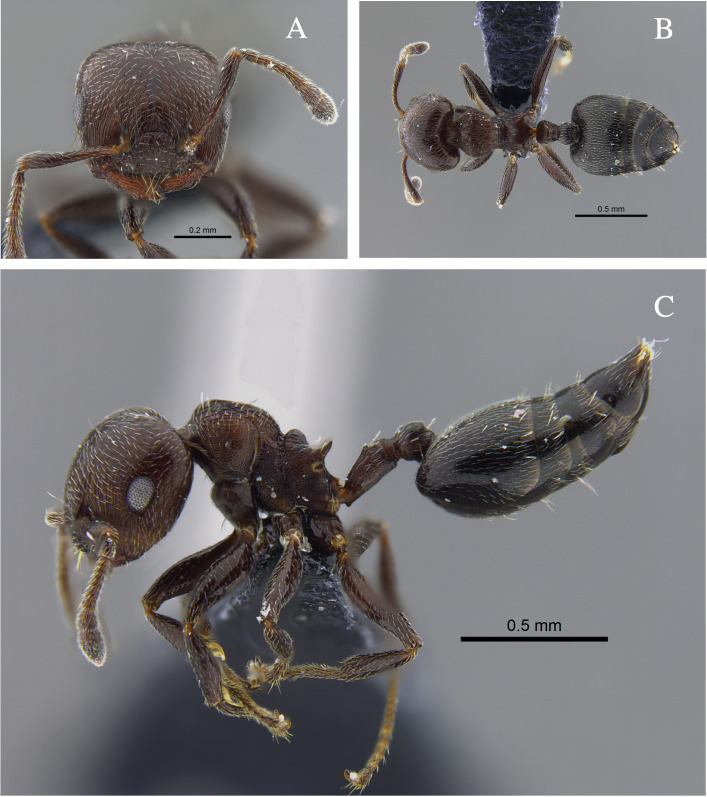
*Crematogaster torosa* worker (CBUMAGENT41928). **A** full-face view; **B** dorsal view; **C** lateral view.

*Crematogaster torosa* (Fig. 22) was attracted to protein and carbohydrate baits in the TDF fragments of the Caribbean Region and was also observed foraging on vegetation and in the leaf litter. In the fragments of Magdalena River Valley and Cauca River Valley regions, it was manually collected while foraging on vegetation, the ground and the litter, whereas in Patía River Valley Region, it was only observed foraging on the ground.

### Crematogaster tdf01

CCF6A6F8-B732-5196-85E8-3A662A517FAB

#### Diagnosis

This is not a formal diagnosis, but distinctive traits are offered, namely: worker with semicircular head, surface smooth and shiny. Mesosoma bearing a distinct V-shaped metanotal groove; propodeal spines short, broad at the base, and slightly divergent. Pronotum with well-defined longitudinal striations. Petiole expanded and globose posteriorly, without ventral tooth. Gaster smooth and shiny. Body pilosity predominantly erect, long and brown, except on legs where the setae are appressed. Colour bicoloured: gaster dark brown, remainder of body light brown. This morphospecies can be distinguished from other *Crematogaster* of the *limata* group by its combination of a semicircular head with a smooth and shiny surface, a pronounced V-shaped metanotal groove, short and broad propodeal spines and a globose petiole lacking a ventral tooth.

#### Distribution

Sucre

##### Distribution in the TDF

Caribbean Region.

#### Biology

**Figure 23.**
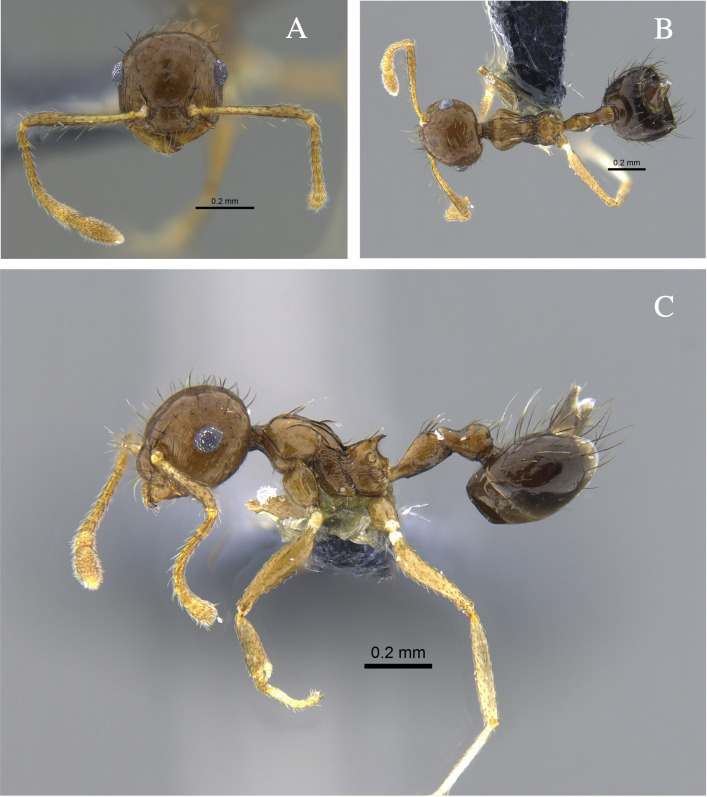
*Crematogaster* tdf01 worker (CBUMAGENT42081). **A** full-face view; **B** dorsal view; **C** lateral view.

*Crematogaster* >tdf01 (Fig. 23) was collected from the leaf litter of dry forest fragments located in the Caracolí Civil Society Nature Reserve (Department Sucre) in the Caribbean Region.

## Analysis

### Species richness of Crematogaster in the Colombian TDF

**Figure 24.**
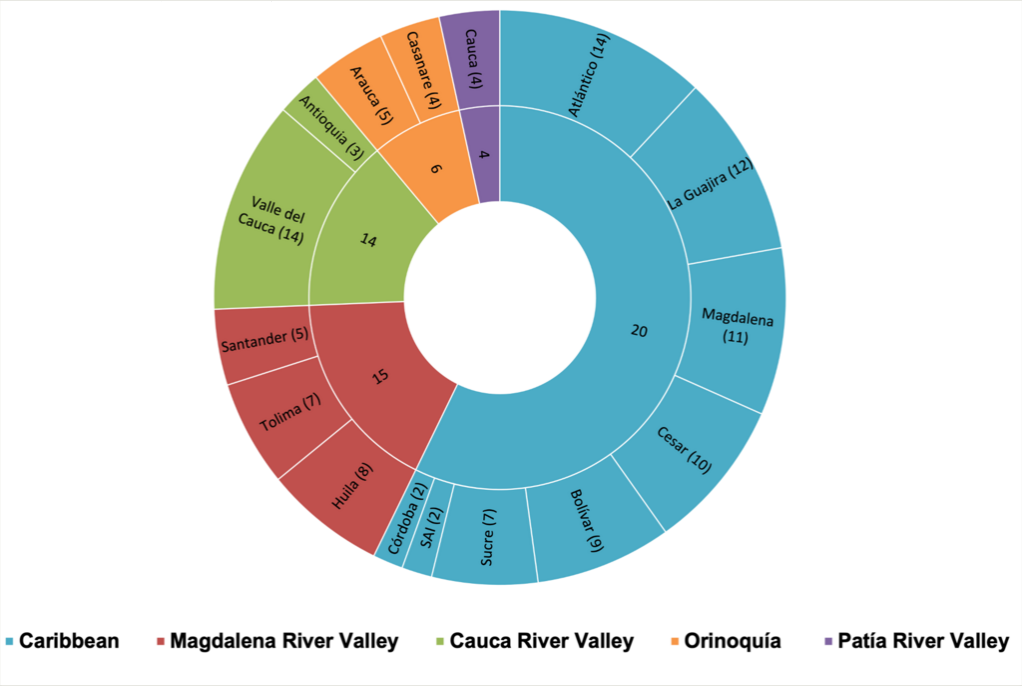
Species richness of *Crematogaster* ants in the Colombian tropical dry forest. Richness values for each region are shown inside, while those in parentheses are the richness for each of the Departments in each respective TDF region. Each TDF region is indicated by a respective colour.

A total of 57,251 specimens were analyzed. Twenty-three *Crematogaster* species were recorded across five Colombian tropical dry forest regions. Species richness varied amongst regions, with the Caribbean showing the highest species richness, with nearly twice as many species as any other region. The Magdalena and Cauca River valleys showed intermediate richness, whereas the Orinoquía and Patía valleys showed considerably lower values (Fig. 24). The Caribbean Region also exhibited the highest number of regionally exclusive species (Table 2), whereas only one exclusive species was recorded in the Cauca River Valley and none in the remaining regions. Pairwise regional overlap was limited and involved only combinations including the Caribbean Region: two species were shared exclusively between the Caribbean Region and Cauca River Valley, four between the Caribbean and Magdalena River Valley and one between the Caribbean and Orinoquía; only one species was shared exclusively between the Cauca and Magdalena River valleys and no other pairwise intersections were detected. At the tri-regional level, two species were shared exclusively amongst the Caribbean, Cauca River Valley and Magdalena River Valley. Broader overlap was observed in higher-order intersections, with four species occurring in the Caribbean, Cauca River Valley, Magdalena River Valley and Orinoquía and two species shared amongst the Caribbean, Cauca River Valley, Magdalena River Valley and Patía River Valley. Only one species was recorded across all five regions (Table 2).

In the Caribbean Region, the Departments of Atlántico, La Guajira, Magdalena and Cesar each recorded more than ten species of *Crematogaster*, whereas the remaining sites contained between two and nine species (Fig. 24). In the Magdalena River Valley Region, Huila and Tolima exhibited the highest numbers of species records. In the Cauca River Valley Region, all *Crematogaster* species recorded (14) were found in Valle del Cauca (Fig. 24). All raw data underlying this study are available in Gutiérrez-Martínez and Guerrero (2025) and are provided in Darwin Core format.

### Multivariate structure of the composition of Crematogaster species

**Figure 25.**
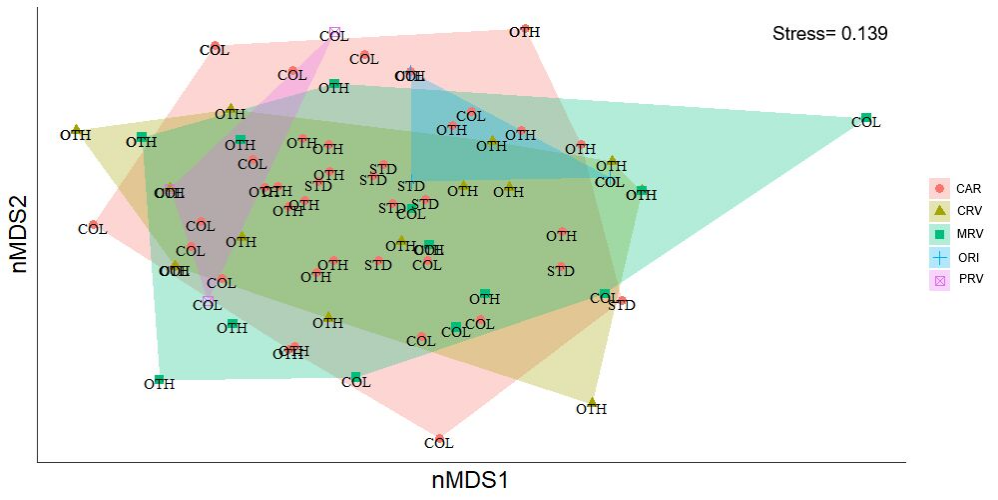
Non-metric multidimensional scaling (nMDS) of *Crematogaster* species from different TDF regions of Colombia, constructed, based on presence/absence data for samples recorded in different locations. The labels above each point (sample) in the multidimensional space correspond to the data source, namely: STD: fieldwork in several regions of tropical dry forest; COL: examination of specimens deposited in national biological collections and museums; and OTH: specimens loaned or donated by other research projects. The convex hulls are enclosing all the points (samples) of the same region: Caribbean (CAR), Cauca River Valley (CRV), Magdalena River Valley (MRV), Orinoquía (ORI) and Patía River Valley (PRV).

The non-metric multidimensional scaling (NMDS) ordination, based on Jaccard dissimilarity, provided an adequate representation of the compositional relationships amongst sampling units across Colombian dry forest regions (stress = 0.139; Fig. 25). Sampling units from the Caribbean (CAR), Cauca River Valley (CRV), Magdalena River Valley (MRV), Orinoquía (ORI) and Patía River Valley (PRV) showed a high degree of overlap in ordination space. No region formed a clearly isolated cluster, suggesting the absence of strong regional segregation in *Crematogaster* species at at the biome scale considered.

PERMANOVA detected a significant overall effect of region on sampling units (*F* = 1.52, *R*² = 0.073, *P* = 0.0106; Suppl. material 2). Pairwise comparisons showed that this regional signal was primarily driven by differences between Caribbean sampling units and those of the Cauca River Valley (*F* = 1.82, *R*² = 0.032, *P* = 0.034) and the Magdalena River Valley (*F* = 1.85, *R*² = 0.033, *P* = 0.019). In contrast, Caribbean sampling units did not differ significantly from those of Orinoquía (*P* = 0.116) or the Patía River Valley (*P* = 0.301). No significant differences were detected amongst most inter-Andean comparisons, including Cauca versus Magdalena (*P* = 0.601), Cauca versus Orinoquía (*P* = 0.087) and Cauca versus Patía (*P* = 0.658).

Data source also had a significant effect on sampling units (*F* = 2.57, *R*² = 0.062, *P* = 0.0001; Suppl. material 2). Pairwise PERMANOVA indicated that sampling units derived from standardized sampling differed significantly from those obtained through other sampling methods (*F* = 2.71, *R*² = 0.052, *P* = 0.003) and from museum and biological collections (*F* = 2.47, *R*² = 0.068, *P* = 0.003). In contrast, sampling units derived from museum collections did not differ significantly from those obtained using other non-standardized methods (*P* = 0.135).

The interaction between region and data source was marginally non-significant (*P* = 0.0676) and residual variation accounted for most of the total variance (*R*² = 0.83), indicating substantial heterogeneity in species composition within regions.

To evaluate whether PERMANOVA results were influenced by differences in multivariate dispersion, we assessed the homogeneity of dispersions using betadisper, based on Jaccard distances. No significant differences in dispersion were detected amongst regions (ANOVA: F₄,₇₃ = 5.26, p = 0.0009; permutation test, 999 permutations, p = 0.002; Suppl. material 1), indicating that the observed PERMANOVA results primarily reflect differences in centroid location rather than unequal within-group variability. Dispersion patterns were broadly comparable amongst regions, despite high internal heterogeneity.

### Distribution of Crematogaster species across Colombian TDFs

**Figure 26.**
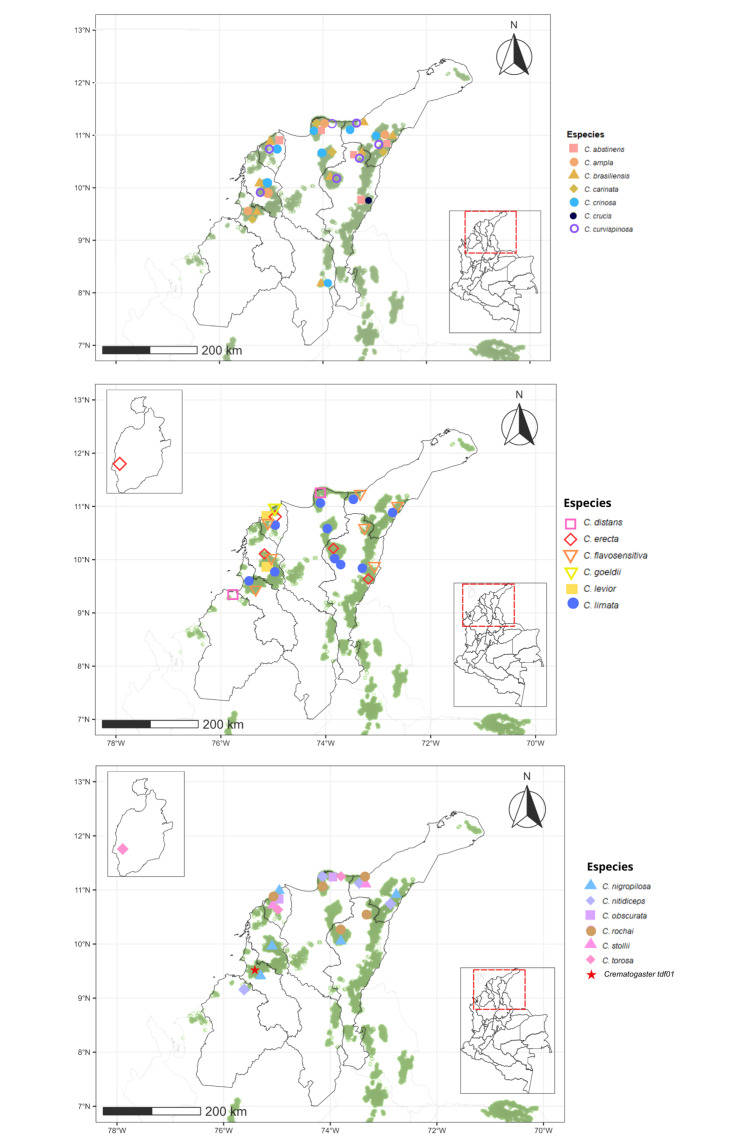
Distributional maps of the *Crematogaster* species in the Caribbean tropical dry forest. On the map of Colombia, the red dashed box indicates the Caribbean Region. An enlarged view on the left shows Departmental boundaries, remaining tropical dry forest areas (green shapes) and occurrence records of *Crematogaster* species across the region. In the middle and bottom map, a solid-line box indicates Providencia Island.

**Figure 27.**
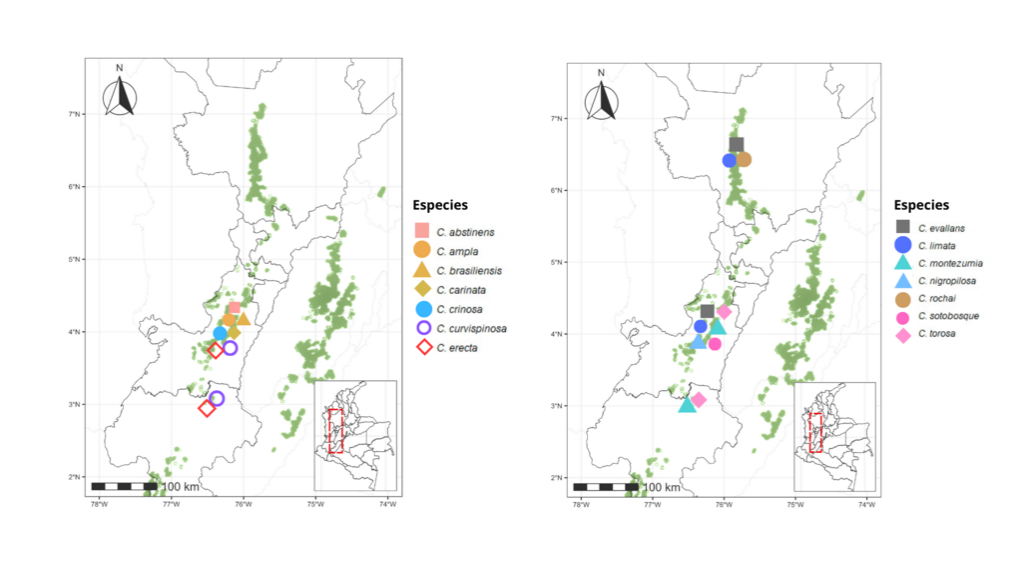
Distributional maps of the *Crematogaster* species in the Cauca River Valley tropical dry forest. On the map of Colombia, the red dashed box indicates the Cauca River Valley Region. An enlarged view on the left shows Departmental boundaries, remaining tropical dry forest areas (green shapes) and occurrence records of *Crematogaster* species across the region.

**Figure 28.**
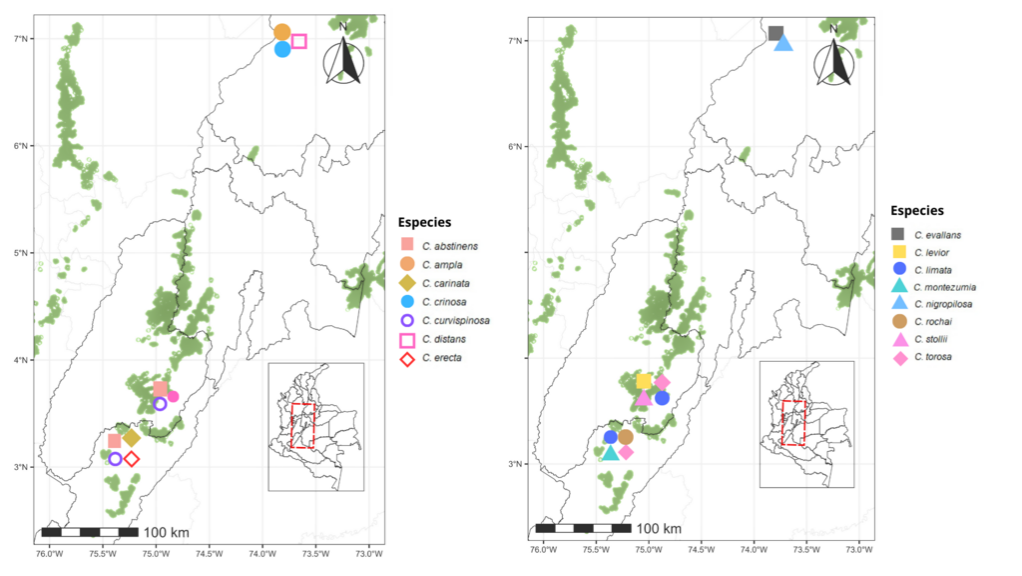
Distributional maps of the *Crematogaster* species in the Magdalena River Valley tropical dry forest. On the map of Colombia, the red dashed box indicates the Magdalena River Valley tropical region. An enlarged view on the left shows Departmental boundaries, remaining tropical dry forest areas (green shapes) and occurrence records of *Crematogaster* species across the region.

**Figure 29.**
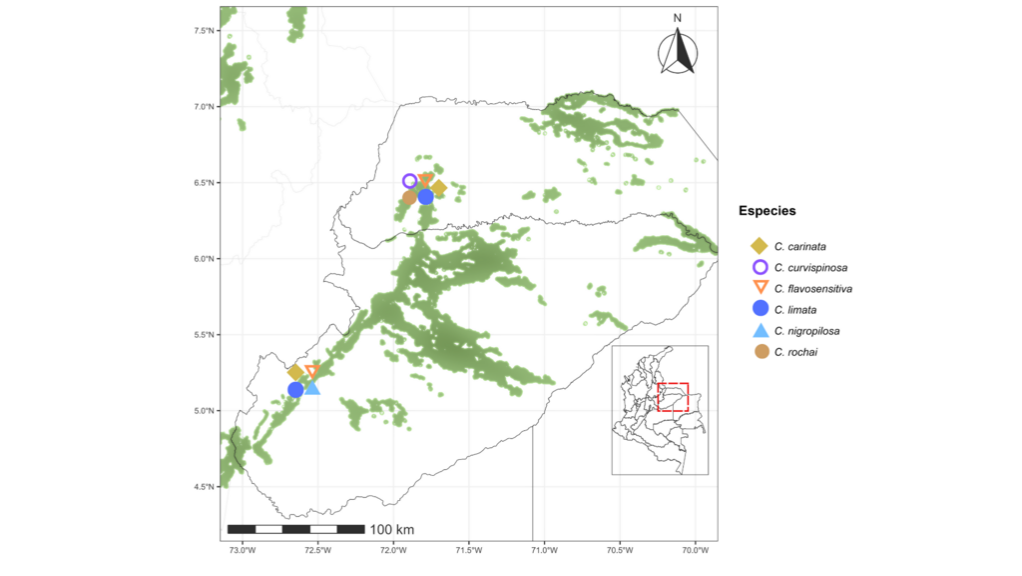
Distributional map of the *Crematogaster* species in the Orinoquía Region tropical dry forest. On the map of Colombia, the red dashed box indicates the Orinoquía tropical region. An enlarged view on the left shows Departmental boundaries, remaining tropical dry forest areas (green shapes) and occurrence records of *Crematogaster* species across the region.

**Figure 30.**
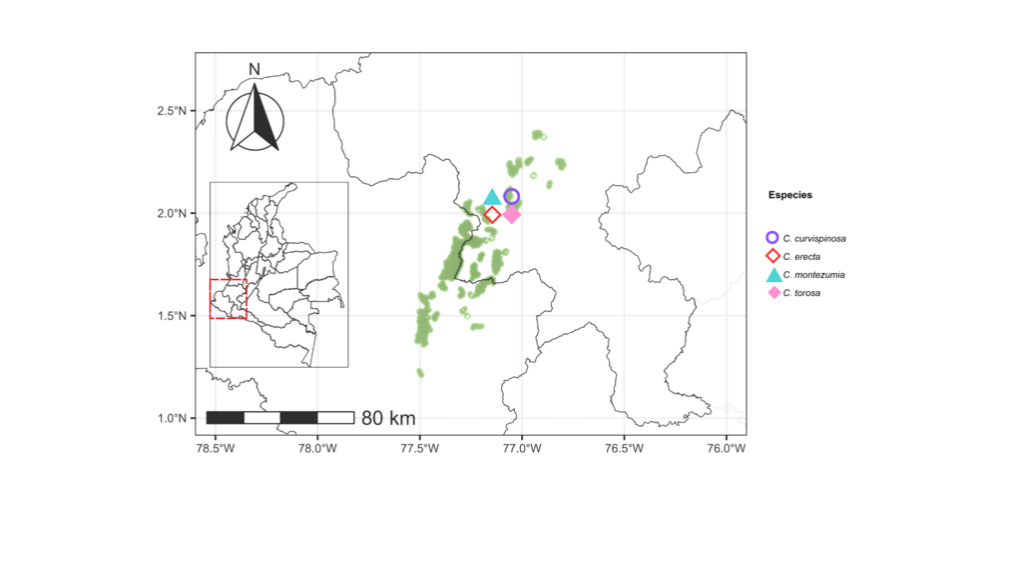
Distributional map of the *Crematogaster* species in the Patia Valley Region tropical dry forest. On the map of Colombia, the red dashed box indicates the Patia Valley tropical region. An enlarged view on the left shows Departmental boundaries, remaining tropical dry forest areas (green shapes) and occurrence records of *Crematogaster* species across the region.

Within the Caribbean Region, which harbours 20 species, seven (*C. brasiliensis*, *C. carinata*, *C. crinosa*, *C. curvispinosa*, *C. limata*, *C. flavosensitiva* and *C. rochai*) occur in four to six Departments. In contrast, *C. crucis*, *C. distans*, *C. levior* and *C. stollii* were restricted to one or two Departments, representing the narrowest distributions in the Caribbean Region. In the Cauca River Valley, five species (*C. curvispinosa*, *C. erecta*, *C. limata*, *C. montezumia*, *C. torosa*) show a wide distribution within the TDF. In the Magdalena River Valley tropical dry forest, only *Crematogaster abistens*, *C. curvispinosa*, *C. limata* and *C. torosa* were found throughout the region. *Crematogaster carinata* and *C. limata* were found in two Departments of the Orinoquía Region. While, in the Patia Valley region, all four species were recorded for only one department (i.e. Cauca) included in this study. Notably, *C. curvispinosa* was the only species recorded in all five regions analyzed (Figs 26, 27, 28, 29, 30).

## Discussion

This study is the first to focus exclusively on documenting the composition and distribution of *Crematogaster* ants in the Tropical Dry Forest of Colombia. Twenty-three *Crematogaster* species were recorded from the TDF of Colombia, representing more than 80% of the genus known in the country (Pedraza and Fernández 2019). *Crematogaster goeldii* is reported for the first time from Colombia, raising the total to 28 species. Geographic ranges of 15 species are extended to additional Departments, confirming the TDF as an important reservoir of *Crematogaster* diversity.

The combined nMDS and Permanova results indicate that sampling units across Colombian dry forests are characterized by high compositional overlap, accompanied by weak, but statistically detectable regional differentiation. The acceptable stress value of the nMDS (0.139) supports the interpretation of broad-scale community patterns, while the ordination topology is consistent with the low proportion of variance explained by region in Permanova results. Pairwise regional comparisons reveal that significant differences are restricted to contrasts involving the Caribbean Region and the two major inter-Andean valleys (Cauca and Magdalena). This suggests that regional differentiation in *Crematogaster* species composition is not pervasive across the biome, but driven by a limited number of regional contrasts with small effect sizes. The broader dispersion of assemblages from the Magdalena River Valley may indicate higher internal heterogeneity in species composition, potentially associated with its latitudinal extent, environmental gradients or historical connectivity with adjacent dry forest regions. In contrast, the tighter clustering of Caribbean and Cauca River Valley assemblages suggests more homogeneous species pools or stronger ecological filtering within these regions. The absence of significant differences involving Orinoquía and the Patía River Valley further supports the view of Colombian dry forests as a largely connected biogeographic system for widespread *Crematogaster* species. On the other hand, the extensive overlap observed amongst Caribbean, inter-Andean valleys (Cauca and Magdalena), Orinoquía and Patía River Valley assemblages likely reflects the ecological generalism of many *Crematogaster* species and their capacity for a wide range of ecological conditions in the TDF biome. This pattern is consistent with the genus’ known tolerance to environmental heterogeneity (i.e. exhibited wide niches) and its frequent dominance in Neotropical habitats (Longino 2003). Furthermore, this pattern is likely related to biological traits such as high dispersal capacity of reproductive castes (Hamidi et al. 2017) and frequent polygyny (Tsuji and Tsuji 1996).

A relevant aspect regarding the trend of our results has to do with the origin of the raw data used. Data sources explained a comparable proportion of variance to region and showed consistent pairwise differences involving standardised sampling. This highlights the influence of sampling design, effort and detectability on inferred sampling units composition and cautions against interpreting all compositional differences as ecological in origin. Nevertheless, the lack of significant interaction between region and data source indicates that regional patterns are broadly consistent across data origins, supporting the integration of heterogeneous datasets for macroecological analyses. Likewise, the dominance of residual variance underscores the importance of local-scale ecological processes, habitat heterogeneity and stochastic factors in structuring *Crematogaster* species groups. Together, these results indicate that regional differentiation exists, but is moderate and that methodological and local ecological factors play a central role in shaping observed patterns of community composition.

Our results should be interpreted considering limitations associated with integrating heterogeneous data sources. The significant effect of data source detected by Permanova (R² = 0.062, P = 0.0001) indicates that differences in sampling design, effort and detectability amongst standardized surveys, museum collections and non-standardized datasets contribute to variation in sampling units composition. However, the lack of significant interaction between region and data source (P = 0.0676) suggests that broad regional patterns are consistent across data origins, supporting the use of integrated datasets for macroecological inference. The high proportion of unexplained variance (residual R² = 0.83) reflects substantial within-region heterogeneity and the influence of local ecological factors not captured at the regional scale. Consequently, regional differences in sampling units composition should be interpreted as moderate and probabilistic rather than sharply delimited. While presence–absence data reduce abundance-related biases (Joseph et al. 2006, Estrada and Arroyo 2012), they may obscure finer-scale gradients in dominance or functional structure (Newbold et al. 2012). Despite these limitations, the concordance between NMDS ordination and PERMANOVA results indicates that the main compositional patterns reported here are robust to methodological heterogeneity and suitable for broad-scale ecological interpretation.

The tropical dry forest in Colombia faces a variety of challenges that differ amongst regions. The Cauca and Patía River valleys show severe fragmentation and vegetation loss (Pizano et al. 2017), whereas the Caribbean and Orinoquía regions retain relatively extensive dry-forest remnants, the country’s four national parks dedicated exclusively to the protection of TDF (González-M et al. 2020). The Caribbean, containing 41% of Colombia’s remaining TDF (García and González-M 2019), harbours the highest *Crematogaster* richness, supported by a greater proportion of protected areas. In contrast, the Magdalena and Cauca valleys maintain only ~ 15% forest cover and support fewer species, although sampling intensity and inclusion of well-preserved sites could be influencing these patterns.

The distribution pattern of *Crematogaster* ants in Colombia’s TDF may also be influenced by the specific characteristics of each region. The Caribbean stands out for its microhabitat diversity, dense vegetation and relatively low anthropogenic disturbance, factors that favour co-existence of multiple ant species (Pizano et al. 2014a, Ramos-Ortega et al. 2022). In contrast, the homogeneous and heavily altered vegetation of the Cauca and Patía valleys reduces resources for arboreal ants such as *Crematogaster*. The stable, humid climate of the Caribbean may further facilitate colonization compared to the more variable conditions of the Magdalena Valley. In the Orinoquía, low species richness likely reflects both limited sampling and insufficient use of arboreal-focused methods, as indicated by results from Casanare where manual collection and canopy sampling proved critical. Species composition shows that the Cauca and Magdalena valleys share many taxa and all species from the Patía Valley are shared with these two regions, suggesting that vegetation composition, rather than geographic distance, drives similarity (Pizano et al. 2014b).

In this study, the Magdalena and Cauca River valleys were found to share the highest number of *Crematogaster* species, while all species recorded from the Patía Valley are also shared with these two regions. This pattern indicates a high degree of similarity in species composition amongst them. Such a finding is noteworthy considering that *Crematogaster* ants are primarily arboreal. The similarity observed in species composition may be related to dominant tree groups that are widely distributed throughout the Colombian dry forest (Norden et al. 2026). Previous studies by Pizano et al. (2014a), using nMDS analyses, showed that the Magdalena, Cauca and Patía River valleys share similar vegetation composition. This supports the idea that the species composition of *Crematogaster* is more strongly influenced by the availability of arboreal resources than by regional biogeographic differences.

At the species level, *Crematogaster curvispinosa* is the only taxon recorded across all five regions, likely due to its capacity to establish in disturbed habitats and respond rapidly to environmental change (Longino 2003, Chacón-de-Ulloa et al. 2012, Pizano et al. 2017, Achury et al. 2020). In contrast, *Crematogaster sotobosque* appears to be exclusive to the Cauca River Valley Region. This pattern is consistent with previous reports documenting its occurrence in this specific area (Chacón-de-Ulloa et al. 2012, Achury et al. 2020). These findings suggest that *C. sotobosque* may have a geographically restricted distribution in this region, possibly as a consequence of habitat fragmentation driven by agricultural expansion and urban development (Pizano et al. 2017). Such processes could be creating ecological barriers that limit the dispersal of its populations to new areas. Similarly, the results indicate that *Crematogaster crucis* seems to have a distribution more restricted to the TDF of the Caribbean Region; however, further sampling and taxonomic studies are needed to confirm and clarify its distribution range.

Our findings suggest that current distribution patterns of *Crematogaster* in Colombia may reflect uneven sampling efforts amongst regions. The Caribbean, Cauca and Magdalena valleys have been more thoroughly surveyed (Armbrecht and Chacón-de-Ulloa 1999, Domínguez et al. 2008, Diaz et al. 2009, Simanca et al. 2013, Camargo-Vanegas and Guerrero 2020), whereas the Patía Valley and Orinoquía remain poorly explored. Taken together, the limitations reported here suggest that the observed regional differentiation in *Crematogaster* species composition should be interpreted as moderate and probabilistic rather than deterministic. Future studies would benefit from increased spatial replication within regions, greater standardization of sampling protocols and the integration of environmental covariates to better disentangle ecological signals from methodological effects. Nevertheless, the concordance between the multivariate analyses results indicates that the main compositional patterns reported here are robust to data heterogeneity and provide a reliable overview of *Crematogaster* species composition structure structure across Colombian dry forests.

From a broader perspective, the weak regional differentiation and high compositional overlap observed across regions have important implications for understanding connectivity in Colombian dry forests. Despite the severe historical and ongoing fragmentation of this biome, *Crematogaster* assemblages appear broadly similar across regions, suggesting effective ecological connectivity at spatial scales relevant for widespread and ecologically flexible ant lineages. This does not imply the absence of fragmentation effects, but rather indicates that such effects may be expressed primarily through local extinctions, changes in species dominance or functional reorganization, rather than through strong regional turnover in species composition. These patterns highlight the importance of habitat structure and resource availability in shaping *Crematogaster* assemblages across TDF regions.

## Supplementary Material

XML Treatment for Crematogaster abstinens

XML Treatment for Crematogaster ampla

XML Treatment for Crematogaster brasiliensis

XML Treatment for Crematogaster carinata

XML Treatment for Crematogaster crinosa

XML Treatment for Crematogaster crucis

XML Treatment for Crematogaster curvispinosa

XML Treatment for Crematogaster distans

XML Treatment for Crematogaster erecta

XML Treatment for Crematogaster evallans

XML Treatment for Crematogaster flavosensitiva

XML Treatment for Crematogaster goeldii

XML Treatment for Crematogaster levior

XML Treatment for Crematogaster limata

XML Treatment for Crematogaster montezumia

XML Treatment for Crematogaster nigropilosa

XML Treatment for Crematogaster nitidiceps

XML Treatment for Crematogaster obscurata

XML Treatment for Crematogaster rochai

XML Treatment for Crematogaster sotobosque

XML Treatment for Crematogaster stollii

XML Treatment for Crematogaster torosa

XML Treatment for Crematogaster tdf01

E4327A20-0816-54DC-8207-75F1EA80D367 10.3897/BDJ.14.e176466.suppl1 Supplementary material 1 The multivariate dispersion of Crematogaster species composition differed significantly amongst the five tropical dry forest regions in Colombia Data type Multivariate scatter plot of sampling units relative to *Crematogaster* species composition Brief description The betadisper ordination revealed marked contrasts in within-region compositional variability, with some regions showing tightly clustered sites around their centroid, whereas others exhibited broader dispersion. File: oo_1520890.png https://binary.pensoft.net/file/1520890 Gutiérrez-Martínez, D., Ramos-Ortega L. M., Guerrero, R. J.

6D061BDE-8198-5F91-A396-0B78F447AE97 10.3897/BDJ.14.e176466.suppl2 Supplementary material 2 Permutational Multivariate Analysis of Variance using region and data source as explanatory factors and post-hoc pairwise multiple comparisons using the adonis2 function in R Data type PERMANOVA and post-hoc pairwise multiple comparisons results Brief description We show the results of the Permutational Multivariate Analysis of Variance and post-hoc pairwise multiple comparisons using the adonis2 function from the vegan package in R. File: oo_1521073.xlsx https://binary.pensoft.net/file/1521073 Gutiérrez-Martínez D., Ramos-Ortega, L. M., Guerrero, R. J.

## Figures and Tables

**Table 1.** General characteristics of the six tropical dry forest regions in Colombia. Information adapted from different sources (Pizano et al. 2014a, Pizano et al. 2014b, Pizano et al. 2017).

TDF Region	Area (km^2^)	Geographic location in Colombia Colombia	Departments distributed throughout the region	Generalized floristic composition
Caribbean	3.9430	North	Atlántico, Bolívar, Cesar, Córdoba, La Guajira, Magdalena, Sucre and San Andrés Island, Providencia and Santa Catalina	The most distinguished tree species in these region are *Terminalia catappa*, *Chrysobalanus icaco*, *Cordia dentata*, *Cedrela odorata*, and *Gliricidia sepium*. There are also large areas with an exotic species, *Tectona grandis*.
Norandina>	1.8470	Central-East	Santander and Norte de Santander	The vegetation in the Norandina> region is determined by the aridity and topography of the Chicamocha River canyon. Subxerophytic formations with thorny scrub and bushes predominate. Endemic species such as *Cavanillesia chicamochae*, *Zamia encephalartoides*, *Melocactus pescaderensis*, and *Melocactus guanensis* stand out.
Orinoquía	0.777	East	Arauca, Casanare, Meta and Vichada	The Eastern Plains are very similar to the Caribbean BST, where we can find species such as *Cecropia peltata*, *Ceiba pentandra*, *Crateva tapia*, *Cordia bicolor*, among others.
Patia River Valley	0.248	South	Cauca and Nariño	This region has vegetation defined by the coverage and height of the trees. The most common species are *Citharexylum kunthianum*, *Pithecelobium dulce*, *Coutarea hexandra*, and *Zanthoxylum caribaeum*, which characterize the vegetation of dense and open forests.
Cauca River Valley	0.312	Southwest	Antioquia, Caldas, Quindío, Risaralda and Valle del Cauca	The most distinctive species in this region are epiphytes such as Orchidaceae and Bromeliaceae. Species such as *Roystonea regia*, *Guadua angustifolia*, and *Abies balsamea* stand out, among others.
Magdalena River Valley	0.268	Center	Boyacá, Cundinamarca, Huila and Tolima	The predominant arboreal species identified in the area include *Anacardium excelsum*, *Attalea butyracea*, *Guazuma ulmifolia*, and *Guarea kunthiana*, among others. Additionally, the presence of various climbing plants, including lianas, contributes to the structural complexity and biodiversity of the local ecosystem.

**Table 2.** Presence–absence matrix of *Crematogaster* species across five tropical dry forest regions of Colombia.

Species name	Caribbean	Cauca River Valley	Magdalena River Valley	Orinoquía	Patía River Valley
*Crematogaster abstinens*	1	1	1	0	0
*Crematogaster ampla*	1	1	1	0	0
*Crematogaster brasiliensis*	1	1	0	0	0
*Crematogaster carinata*	1	1	1	1	0
*Crematogaster crinosa*	1	0	1	0	0
*Crematogaster crucis*	1	0	0	0	0
*Crematogaster curvispinosa*	1	1	1	1	1
*Crematogaster distans*	1	0	1	0	0
*Crematogaster erecta*	1	1	1	0	1
*Crematogaster evallans*	0	1	1	0	0
*Crematogaster flavosensitiva*	1	0	0	1	0
*Crematogaster goeldii*	1	0	0	0	0
*Crematogaster levior*	1	0	1	0	0
*Crematogaster limata*	1	1	1	1	0
*Crematogaster montezumia*	0	1	1	0	1
*Crematogaster nigropilosa*	1	1	1	1	0
*Crematogaster nitidiceps*	1	0	0	0	0
*Crematogaster obscurata*	1	1	0	0	0
*Crematogaster rochai*	1	1	1	1	0
*Crematogaster sotobosque*	0	1	0	0	0
*Crematogaster stollii*	1	0	1	0	0
*Crematogaster torosa*	1	1	1	0	1
*Crematogaster* TDF01	1	0	0	0	0
