# Supplementary figures and images for: Diversity and distribution of acrobat ants, Crematogaster Lund, 1831 (Formicidae, Myrmicinae), in the Colombian tropical dry forest
Source: Biodivers Data J. 2026 Apr 27;14:e176466. doi: 10.3897/BDJ.14.e176466 (PMC13139862; doi:10.3897/BDJ.14.e176466)

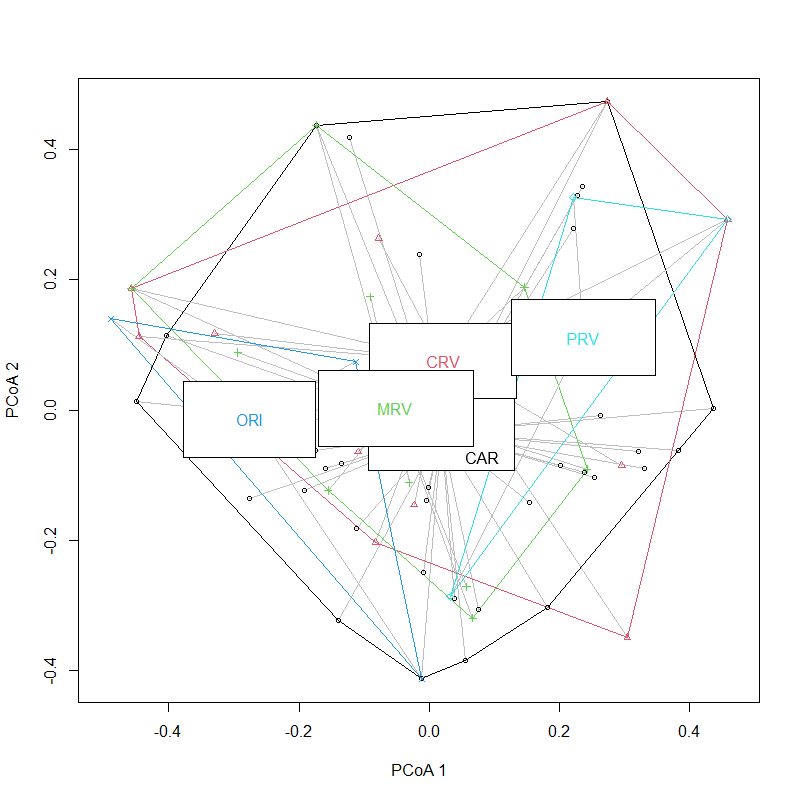

Supplement: Supplementary material 1 — The multivariate dispersion of Crematogaster species composition differed significantly amongst the five tropical dry forest regions in Colombia [file bdj-14-e176466-s001.png]
